# Chemopreventive and therapeutic effects of *Hippophae rhamnoides* L. fruit peels evaluated in preclinical models of breast carcinoma

**DOI:** 10.3389/fphar.2025.1561436

**Published:** 2025-04-30

**Authors:** Dana Dvorska, Dominika Sebova, Karol Kajo, Andrea Kapinova, Emil Svajdlenka, Michal Goga, Richard Frenak, Jakub Treml, Sandra Mersakova, Jan Strnadel, Alena Mazurakova, Ivana Baranova, Erika Halasova, Mariana Brozmanova, Kamil Biringer, Monika Kassayova, Zuzana Dankova, Karel Smejkal, Slavomir Hornak, Jan Mojzis, Vladimira Sadlonova, Dusan Brany, Martin Kello, Peter Kubatka

**Affiliations:** ^1^ Biomedical Centre Martin, Jessenius Faculty of Medicine, Comenius University in Bratislava, Martin, Slovakia; ^2^ Department of Pharmacology, Faculty of Medicine, P. J. Šafárik University, Košice, Slovakia; ^3^ Department of Pathology, St. Elisabeth Oncology Institute, Bratislava, Slovakia; ^4^ Department of Natural Drugs, Faculty of Pharmacy, Masaryk University, Brno, Czechia; ^5^ Department of Botany, Institute of Biology and Ecology, Faculty of Science, P. J. Safarik University, Kosice, Slovakia; ^6^ Department of Molecular Pharmacy, Faculty of Pharmacy, Masaryk University, Brno, Czechia; ^7^ Department of Anatomy, Jessenius Faculty of Medicine, Comenius University in Bratislava, Martin, Slovakia; ^8^ Department of Pathological Physiology, Jessenius Faculty of Medicine, Comenius University in Bratislava, Martin, Slovakia; ^9^ Biobank for Cancer and Rare Diseases, Jessenius Faculty of Medicine, Comenius University in Bratislava, Martin, Slovakia; ^10^ Clinic of Obstetrics and Gynecology, Jessenius Faculty of Medicine, Comenius University in Bratislava, Martin, Slovakia; ^11^ Department of Animal Physiology, Institute of Biology and Ecology, Faculty of Science, P. J. Safarik University, Kosice, Slovakia; ^12^ Small Animal Clinic, University of Veterinary Medicine and Pharmacy, Kosice, Slovakia; ^13^ Department of Microbiology and Immunology, Jessenius Faculty of Medicine, Comenius University in Bratislava, Martin, Slovakia; ^14^ Laboratory of Experimental and Clinical Regenerative Medicine, Small Animal Clinic, University of Veterinary Medicine and Pharmacy, Kosice, Slovakia

**Keywords:** breast carcinoma, cancer stem cells, epigenetics, *Hyppophae rhamnoides* L., chemoprevention, rodent models, therapy modulation

## Abstract

**Background:**

Cancer remains a major global health challenge, necessitating innovative prevention and treatment approaches. Certain plants, adapted to specific environments, may exhibit bioactive properties with potential anticancer applications.

**Hypothesis:**

Seaberry (*Hippophae rhamnoides L.*) fruit peels may exert anticancer effects in breast carcinoma (BC) models through the additive or synergistic actions of their unique secondary metabolites.

**Methods:**

*H. rhamnoides* fruit peel extracts were analyzed using the LC-DAD-MS and LC-DAD techniques to profile the content of carotenoids and flavonoids, respectively. The preclinical study evaluated seaberry fruit peel extracts in BC models: (1) a syngeneic 4T1 mouse breast adenocarcinoma model (triple-negative), (2) a rat model of chemically induced mammary carcinogenesis, and (3) *in vitro* studies with MCF-7 (hormone receptor-positive) and MDA-MB-231 (triple-negative) BC cell lines.

**Results:**

LC-DAD-MS and LC-DAD analyses identified dominant metabolites, including isorhamnetin, quercetin glycosides, kaempferol glycosides, catechin, zeaxanthin, and lutein. In the 4T1 mouse model, seaberry treatment resulted in a significant, dose-dependent reduction in tumor volume (43% and 48% compared to controls) and a decrease in the mitotic activity index. Serum cytokine analysis showed dose-dependent reductions in IL-6, IL-10, and TNF-α. In the rat chemopreventive model, high-dose seaberry improved cancer prognosis by reducing the ratio of poorly differentiated tumors and increasing caspase-3 and Bax expression while decreasing Ki-67 and malondialdehyde levels. Both treatment doses elevated the Bax/Bcl-2 ratio and reduced the expression of cancer stem cell markers CD44, EpCam, and VEGF compared to controls. Epigenetic analyses revealed histone modifications (H4K16ac, H4K20me3) and altered methylation of tumor-suppressor genes (PITX2, RASSF1, PTEN, TIMP3). Microarray analysis (758 miRNAs) identified beneficial changes in nine oncogenic/tumor-suppressive miRNAs, including miR-10a-5p, miR-322-5p, miR-450a-5p, miR-142-5p, miR-148b-3p, miR-1839-3p, miR-18a-5p, miR-1949, and miR-347. *In vitro*, ethanolic seaberry extract conferred partial resistance to cisplatin-induced cytotoxicity in MCF-7 and MDA-MB-231 cells at IC_50_ concentrations.

**Conclusion:**

This study of *H. rhamnoides* in rodent BC models shows promising data but requires rigorous, long-term validation. Integrating plant-based nutraceuticals into oncology necessitates precise cancer-type profiling and patient stratification for effective personalized treatments.

## 1 Introduction

Epidemiological studies consistently demonstrate a strong association between diets abundant in fruits, vegetables, herbs, tea, legumes, and whole grains and a reduced risk of developing chronic diseases, including cancer (Marino et al., 2024; Nagy et al., n.d.). Emerging evidence highlights the pivotal role of phytochemicals in delivering antioxidant and anti-inflammatory effects that contribute to genoprotection. Phytochemicals are increasingly recognized as modulators of key signaling pathways involved in cellular processes such as apoptosis, proliferation, angiogenesis, stem cell regulation, metastasis, differentiation, and epigenetic modifications (Choudhari et al., 2020; Di Napoli et al., 2023; Rudzińska et al., 2023).

*Hippophae rhamnoides* L. (commonly known as seaberry, seabuckthorn, or Siberian pineapple) is a deciduous plant belonging to the genus *Hippophae* within the family Elaeagnaceae (Sharma and Kalkal, 2018). Recognized globally for its pharmaceutical and nutraceutical applications, seaberry is now cultivated extensively across various regions worldwide (Masoodi et al., 2020). It is notable for its unique composition of bioactive compounds, including phenolic compounds, high levels of vitamin C, unsaturated fatty acids, and phytosterols such as beta-sitosterol (Olas et al., 2018a). Seaberry products, including berries, juices, jams, and oils, are associated with various beneficial properties, including antioxidant, anti-inflammatory, and anticancer effects (Olas et al., 2018a; Jaśniewska and Diowksz, 2021; Dubey et al., 2023). Seaberry has great potential as a cancer therapeutic agent, owing to its antiproliferative activities, apoptosis-inducing properties, immune system enhancement, and ability to alleviate chemotherapy side effects. Seaberry oil, in particular, has been shown to improve kidney and liver function, enhance appetite, and support the overall wellbeing of patients undergoing treatment (Batbold and Liu, 2022; Feng et al., 2023; Mihal et al., 2023). Several *in vitro* studies have further demonstrated the anticancer potential of seaberry. For instance, initial research combining seaberry fruit extract with docetaxel revealed a synergistic anticancer effect in two non-small cell lung cancer cell lines (A549 and H23). The treatment induced caspase-independent autophagy and senescence, accompanied by increased reactive oxygen species (ROS) production, elevated expression of microtubule-associated protein one light chain 3 (LC3), G1-phase cell cycle arrest, enhanced senescence-associated β-galactosidase activity, and increased ERK phosphorylation (Batbold and Liu, 2022). Another study investigated the antiproliferative effects of seaberry fruit extracts on cancer cell lines. The ethyl acetate extract demonstrated the most potent inhibitory activity against Caco-2 cells, while the ethanol: water extract was most effective on Hep G2 cells. Both extracts exhibited dose-dependent antiproliferative effects, with the ethyl acetate extract showing a strong association with enhanced apoptosis (Grey et al., 2010). Furthermore, a study on the lipophilic extract of seaberry in two breast cancer (BC) cell lines—T47D (ER^+^, PR^+^, HER2-) and BT-549 (ER-, PR-, HER2-)—demonstrated concentration-dependent antiproliferative effects. The treatment reduced ROS levels, indicative of antioxidant activity, and induced changes in late-stage apoptotic cells. These findings highlight the proapoptotic properties of seaberry carotenoids against BC cells (Visan et al., 2023).

This research was predicated on the hypothesis that plant-based nutraceuticals, particularly those rich in phytochemicals with potential additive or synergistic effects, can exhibit significant antitumor properties, as demonstrated in our prior studies (Kubatka et al., 2015; 2016b; 2016a; 2017a; 2017b; 2019; 2020a; 2020b; 2024; Dvorska et al., 2024). *H. rhamnoides* (sea buckthorn) fruit peels are a compelling nutraceutical for BC models due to their rich content of bioactive compounds, including carotenoids, polyphenols, and flavonoids, which exhibit significant anticancer activities (Pop et al., 2014; Yang et al., 2016; Ji et al., 2020). The diverse and potent bioactive profile of *H. rhamnoides* offers a multifaceted approach to BC management, distinguishing it from other phytopharmaceuticals and enhancing its potential efficacy in cancer prevention and treatment (Wang et al., 2022; Visan et al., 2023).

The objective was to investigate the anticancer effects of *H. rhamnoides* fruit peels in therapeutic (allograft) and chemopreventive BC animal models. Mechanistic analyses focused on established cancer biomarkers, including those associated with cell death, proliferation, angiogenesis, inflammation, oxidative stress, stem cell regulation, and epigenetic modifications. Histopathological evaluations of tumor samples were also conducted, encompassing parameters such as the ratio of high-to low-grade carcinomas, tumor necrosis ratio, and mitotic activity index. To further elucidate the anticancer mechanisms of *H. rhamnoides* fruit peels, an additional study was conducted on human BC cell lines, specifically the hormone-sensitive MCF-7 and the metastatic triple-negative MDA-MB-231 cells. This study aimed to determine whether the extract could mitigate the cytotoxic effects of cisplatin, thereby providing insights into its potential as a protective agent in BC treatment.

## 2 Materials and methods

### 2.1 Plant material

*Hippophae rhamnoides* fruit peel powder was obtained from the company Zamio (Zamio, Michalovce, Slovakia, Zemplin region). The batch number of the plant material is 092,022.

### 2.2 The examinations of plant secondary metabolites in *Hippophae rhamnoides* extracts

The plant material was processed according to further described procedures. Generally, the fruit peel contains 39.46% of lipophilic *n*-hexane extractable substances and 21.23% of compounds extractable with EtOH: H_2_O 2:1 (v/v), and 39.31% of residuum not extractable with these solvents. To evaluate the *H. rhamnoides* fruit peel in a complex way, we analyzed and determined the content of carotenoids in *n*-hexane extract and catechine and flavonoids in EtOH: H_2_O 2:1 (v/v) extract. For catechine and flavonoids, we utilized LC-DAD-MS, for carotenoids LC-DAD analysis, respectively. All solvents used were of gradient grade or MS quality (VWR Chemicals).

#### 2.2.1 Extraction of H. rhamnoides

Briefly: *H. rhamnoides* fruit peel (2.123895 g) was mechanically disintegrated and extracted on a Soxhlet extractor (40 cycles) with 100 mL of *n*-hexane; the rest was overnight dried at laboratory temperature and subsequently extracted with 100 mL of EtOH: H_2_O 2:1 (v/v) on Soxhlet extractor (40 cycles).

#### 2.2.2 Flavonoid content analysis

The quantitative analysis of flavonoid content was performed utilizing LC-DAD-MS. After filtration of EtOH: H_2_O 2:1 (v/v) extract, one aliquot was used to analyze the content of aglycones (quercetin, kaempferol, and isorhamnetin). The second extract aliquot was subjected to hydrolysis. 1 mL of extract was hydrolyzed by 0.6 mL of diluted HCl [conc. HCl: water 2:8 (v/v)] for 20 min at 100°C and after cooling, it was injected to analyze the content of flavonoglycosides of quercetin, kaempferol, and isorhamnetin. The analyses were done using analytical HPLC-DAD-MS instrument Agilent 1260 chromatographic system (1260 Vial sampler G7129A, 1260 Quat Pump G7111B, 1260 MCT G7116A, 1260 DAD HSG7117C, all Agilent Technologies, Waldbronn, Germany) coupled with MS AB SCIEX Triple Quad 3500 system (Framingham, United States). The column InfinityLab Poroshell 120 EC-C18, 4.6 × 100 mm, 2.7 μm (Agilent), mobile phase consisting of A (0.1% HCOOH and 1 mmol/L HCOONH_4_ in MeOH (HPLC grade, Sigma)) and B (0.1% HCOOH and 1 mmol/L HCOONH_4_ in H2O (HPLC grade, Sigma)) was used. Gradient elution: 0. min 60% of A, 4 min 80% A, 4.5 min 100% A, 8 min 100% A, 8.1 min 60% A, 13 min.60% A. Flow rate 0.5 mL/min. The column block temperature is 30°C, injection volume is 3 μL. The MS quantification was done using ESI MS in negative MRM mode, with two transitions for each analyte. MS conditions: curtain gas N_2_ 25 L/min, temperature 450°C, gas no. 1 50 L/min, gas no. 2 40 L/min, ion spray voltage −4500 V, scan rate 1000 Da/s, solvent delay time 4 min. Compounds were identified by comparing their retention times, UV, and MS profiles with standards. The quantification was carried out by using MS (2 MRM transitions for each compound) and UV scan λ 190–400 nm calibration curves, constructed based on measurements of the corresponding standards. DAD was set to 268 nm (bw 4, reference 450/bw 100) for internal standard chrysin and 367 (bw 6, reference 500/bw 50) for flavonoids. Quercetin, kaempferol, and isorhamnetin glycosides and corresponding aglycones were analyzed (Supplementary Figure S1); external standards were used for calibration. The total content of flavonoids was calculated as hypothetic flavonoglycoside (Mr 756.7 g/mol) from the sum of determined aglycones after hydrolysis of the fruit peel flavonoglycosides.

#### 2.2.3 Catechine content analysis

After filtration of EtOH extract (2.123,895 g/100 mL), 1 μL was injected to analyze the content. Pure catechine (Sigma Aldrich, Germany) was used as a standard to determine the content. Agilent 1260 HPLC quaternary pump and DAD with AB SCIEX 3500 TripleQuad machine were used; Agilent InfinityLab Poroshell 120 EC-C18, 4.6 × 100 mm, particle size 2.7 um with guard column of the same type (5 mm, i.d.). Flow rate 0.3 mL/min, gradient of A (MeOH with 0.1% HCOOH and 1 mmol/L HCOONH_4_) and B (H_2_O with 0.1% HCOOH and 1 mmol/L HCOONH_4_): 0 min 10% of A, 18 min 100% of A; column block temperature 30°C; DAD scan 190–600 nm; MS in negative MRM mode, MRM transitions for catechine 289-245, second 289-122; curtain gas CUR 25, collision gas CAD 8, ion spray voltage IS −4500, nebulizer temperature TEM 450°C, ion source gas 1 G 50, ion source gas 2 40 (Supplementary Figure S2).

#### 2.2.4 Carotenoid content analysis

For analysis, 1 mL of *n*-hexane extract was evaporated by the stream of nitrogen and redissolved in 1 mL of 20% THF in MeOH. Agilent 1260 HPLC quaternary pump and DAD, with Ascentis Express RP-Amide, 150 × 2.1mm, 2.7 µm (Supelco). The mobile phase consisted of A (acetonitrile with 1 mM of HCOONH_4_ and 0.1% of HCOOH) and B (0.16 g HCOONH_4_ and 2.5 mL HCOOH for 2.5 L of water), with a gradient of A in 0. minute 10%, 15. minute 100%, 18. minute 100%, and return to starting conditions in 40. minute. The flow rate was set to 0.3 mL/min, column block temperature 30°C, and the UV detector set to 450 nm (bw 4 for lutein and zeaxanthin and bw 100 for carotenoids, ref. off) (Supplementary Figure S3). The injection volume was 1 μL, and the sampler was set to 20°C. Lutein (Sigma Aldrich, Lot: LRAC6944) and zeaxanthin (USP ref. St. Aztec Marigold extract, Lot: F0L455) solutions were used as standards for analysis to construct the calibration curves.

#### 2.2.5 Extraction of plant material for in vitro testing

Five grams of dried plant material was macerated in 100 mL of solvent (ethanol, methanol, or n-hexane) for 2 h, at a room temperature under continuous stirring with a magnetic stirrer. The aqueous extract was prepared by the same method at a temperature of 80°C. This extraction procedure was repeated three times, and the extracts were pooled. The resulting extracts were filtered and solvents were removed by using a rotary vacuum evaporator. The extracts were then stored at 4°C in a refrigerator until further analysis.

### 2.3 Rodent models

The studies received approval from the Ethical Commission of the Jessenius Faculty of Medicine at Comenius University (Protocol No. EK 1860/2022) and the State Veterinary and Food Administration of the Slovak Republic (accreditation No. Ro-5056-3/2022-220).

#### 2.3.1 Animals

Female BALB/c mice (Velaz, Prague, Czech Republic) aged 10 weeks and weighing between 17 and 19 g and female Sprague-Dawley rats (Charles River Laboratories, Sulzfeld, Germany) aged 5 weeks and weighing between 125 and 140 g were utilized in the experiment. The rodents were accustomed to a controlled vivarium setting with specific conditions such as a 12-h artificial light cycle, a temperature range of 23°C ± 2°C, and a relative humidity range of 40%–60%. They were given *ad libitum* access to a Ssniff^®^ diet (R-Z/M-Z low-phytoestrogen; Soest, Germany) and tap water. Mammary gland cancer in rats was induced by the application of N-nitroso-N-methylurea (NMU, Sigma, Deisenhofen, Germany). The carcinogen was injected intraperitoneally at a single dosage of 50 mg/kg on the 42nd postnatal day. The timing of carcinogen administration during early puberty (postnatal days 40–46) significantly increased the susceptibility to mammary carcinogenesis in Sprague-Dawley rats compared to administration after postnatal day 50. This method of research mirrors the higher risk of BC etiology in premenopausal women. A syngeneic mouse model was used to represent the BC treatment model. Mammary carcinogenesis was induced by subcutaneously injecting 1 × 10^4^ 4T1 cells per animal (mouse mammary adenocarcinoma) into the abdominal mammary gland area.

#### 2.3.2 Diet

The administration of *H. rhamnoides* fruit peel powder (Zamio, Michalovce, Slovakia, Zemplin region) in the allograft mouse model (treatment study) began on the same day as the inoculation of 4T1 cells and continued for 16 days. In rats, *H. rhamnoides* fruit peels administration (chemoprevention study) commenced 1 week before carcinogenesis induction and lasted for 14 consecutive weeks. The fruit peels underwent processing through the “cold pelleting procedure” to be included in the diet. In both animal models, *H. rhamnoides* fruit peels were administered in low concentrations of 3 g/kg–0.3% (w/w) (SEA 0.3) and higher concentrations of 30 g/kg–3% (w/w) (SEA 3). The administration of seaberry (sea buckthorn) at a concentration of 0.3% w/w in our study was based on traditional human consumption patterns, approximately equivalent to 20 g of dried fruit per day: Sea Buckthorn Uses, Benefits & Dosage (n.d.). Given the potential differences in the pharmacokinetics and pharmacodynamics of seaberry’s secondary metabolites between humans and rodents, we incorporated a higher dietary dose of 3% w/w in our rodent studies. This precautionary measure was taken due to the lack of prior publications on seaberry supplementation in rodent BC models. By employing the 3% w/w dosage, we aimed to ensure that any potential oncostatic effects of seaberry were not overlooked by relying solely on the lower dose. A total of 60 mice and 75 rats were randomly assigned to three different experimental groups. The first group served as the control group, with no *H. rhamnoides* in the diet, acting as the blank control, where the background and diet remained unaltered. The second group received *H. rhamnoides* in the diet at a lower dose (SEA 0.3), while the third group received *H. rhamnoides* in the diet at a higher dose (SEA 3).

#### 2.3.3 Animal experiment procedures

Following the fourth day post-inoculation of 4T1 cells in mice, the tumor growth (volume) was monitored three times weekly. Starting from the fifth week after the application of the carcinogen, the rats were palpated weekly to assess the presence, size, and location of each mammary tumor (considered palpable if the tumor diameter exceeded 0.4–0.5 cm). Tumor incidence was calculated as the percentage of animals with tumors in each group, tumor frequency was determined by the total number of tumors observed in all animals within the group, and the latency period was defined as the time from carcinogen administration to the appearance of the first tumor in the animal. Throughout 24 h, the dietary consumption of mice was observed two times, while rats were monitored four times. Consequently, the mean daily quantities of *H. rhamnoides* given to each group of mice and rats were computed. At the end of the experiments, the rodents were humanely euthanized through quick decapitation, and mammary lesions were excised and assessed.

### 2.4 Histopathology of rodent tumor samples

Each tissue sample from rodent (mouse and rat) carcinoma underwent routine formalin fixation and paraffin embedding. For the mouse model, each tumor’s average tumor area and average necrosis area were calculated. Subsequently, the necrosis ratio was determined as the ratio of the average necrosis area to the average tumor area. The tumor area and necrosis extent were assessed on histological slides. The area of the tumor was calculated based on its shape–circular/the area was determined as πr^2^ (*r* = radius)/, ellipse/π × *a* x *b* (*a*, *b* = semi-axes)/, rectangle/*a* × *b* (*a*, *b* sides of the rectangle)/, or trapezoid (*a* + *c*) c v/2. If the tumor has grown to the size of one of the high-power fields (HPFs) (e.g., with ×4 objective), the area was determined based on this microscopic field. The contours of the necrosis were outlined in the histological slide and the area of the necrosis was usually defined as their extent according to the size of the HPF, or multiple of the area of the HPF (with objective ×4 = 23.76 mm^2^, with objective ×10 = 3.8 mm^2^), with objective ×20 = 0.95 mm^2^). The smallest evaluable necrosis was its extent in one HPF (0.24 mm^2^; i.e., at magnification ×400 with a diameter of the field of view 0.5 mm). If necrosis was smaller than the HPF, it was evaluated as punctiform.

A comprehensive microscopic evaluation was conducted on tumor samples collected from rats. Initially, at low magnification of ×40, the overall tumor structure was observed, focusing on growth microarchitectural features such as the proportion of glandular and solid components and necrosis for histological grading. Subsequently, detailed cellular characteristics including the degree of cellular atypia and the number of mitotic figures were examined at higher magnifications of ×100 and ×200. The mitotic score, applicable to both rats and mice, was calculated based on the number of mitotic figures observed in 10 consecutive HPFs in the most mitotically active area of the tumor. Only identifiable mitotic figures were tallied, while hyperchromatic, karyorrhetic, or apoptotic nuclei were excluded. In rare instances where the total tumor area from mice was less than the sum of 10 HPFs, the number of mitoses in five HPFs was assessed and then doubled.

The standardized classification criteria for rat mammary tumors included sub-division into low-grade or high-grade tumors. The categorization criteria utilized in the standard diagnostic classification method involved solidization, index of mitotic activity, and necrosis. Solidization was determined if >30% of the tumor sample exhibited solid growth, a high index of mitotic activity was noted if ≥10 mitoses were observed in 10 HPFs, and necrosis was confirmed by the presence of comedo (not infarct). High-grade carcinomas were identified by having ≥2 positive criteria, while low-grade carcinomas had ≤1 positive criterion. In mice tumor samples, the mitotic activity index and tumor area/necrosis ratios were evaluated.

### 2.5 Immunohistochemistry of rodent tumor samples

The mammary tumor sample selection for immunohistochemical analysis was based on specific criteria, ensuring the representation of vital tumor epithelial components without any regressive changes such as necrosis. The detection of markers for the mechanistic study was carried out using an indirect immunohistochemical method on whole paraffin sections with commercially available rat-specific antibodies from various suppliers (Thermo Fisher Scientific, Rockford, IL, United States; Santa Cruz Biotechnology, Paso Robles, CA, United States; GeneTex, Irvine, CA, United States; Dako, Glostrup, Denmark; Boster Biological Technology, Pleasanton, CA, United States; Bioss, Woburn, MA, United States; Abcam, Cambridge, MA, United States). The immunohistochemical staining was performed according to the manufacturer’s recommendations using Autostainer Link 48/Hermes. The concentrations of each primary antibody used were as follows: Bax 1:200 (catalogue No. sc-526); Bcl-2 1:200 (sc-492); cleaved caspase-3 1:500 (ab2302); Ki-67 1:50 (M7248 01); VEGFA 1:150 (sc-57496); VEGFR-2 1:80 (sc-6251); MDA 1:1,000 (ab6463); EpCam 1:160 (ab71916); ALDH1A1 1:500 (pa532127); CD133 1:150 (ab19898); CD44 1:200 (pa1021-2); CD24 1:200 (gtx37755); H3K4m3 1:500 (ab8580); H3K9m3 1:400 (ab8898); H4K16ac 1:200 (ab109463), H4K20m3 1:300 (ab9053). The primary antibodies were visualized using diaminobenzidine tetrahydrochloride as a substrate and the EnVision secondary staining system (Dako North America, Carpinteria, CA, United States, cat. No. K060911). Negative controls were included by omitting primary antibodies. The expression of selected antigens was evaluated through precise morphometric analysis following immunohistochemical detection, with initial screening and microscopic analysis performed at magnifications of digital images ×400 using an Olympus BX41N microscope. The protein expression quantification was determined by calculating the average percentage of antigen-positive areas in standard fields (0.5655 mm^2^) of hot spot areas in tumor cells. Three hot spots were examined per tumor sample using the morphometric method. Digital image analysis was conducted with QuickPHOTO MICRO software, version 3.0 (Promicra, Prague, Czech Republic). The values obtained were then compared between tumor tissue samples from experimental groups fed with seaberry (SEA 0.3 and SEA 3) and untreated (control) tumor tissue samples. A total of 60 tumor samples were assessed for each marker (960 tumor slides for 16 markers).

### 2.6 Analysis of miRNA expression

#### 2.6.1 Isolation of total RNA

Approximately 30 mg of tissue was excised from samples stabilized in RNAlater. The tissue was then combined with 700 µL of QIAzol Lysis Reagent (Qiagen, Hilden, Germany) and sterile stainless-steel beads (5 mm in diameter, Qiagen). Homogenization was performed using the TissueLyser II (Qiagen) at 50 Hz for 5 min. Total RNA was subsequently isolated from the homogenized samples using the miRNeasy Mini Kit (Qiagen), following the manufacturer’s protocol. The purified RNA was eluted in RNase-free water and stored at −80°C until further use.

#### 2.6.2 Assessment of RNA quality and quantity

The concentration and purity of the isolated total RNA were initially determined spectrophotometrically using a NanoDrop OneC device (Thermo Fisher Scientific, Waltham, MA, United States). Subsequently, RNA quality and concentration were evaluated using the Agilent 2100 Bioanalyzer with the RNA 6000 Nano Kit (Agilent Technologies, Santa Clara, CA, United States). Only samples meeting the following criteria were selected for further analyses: RNA integrity number (RIN) ≥7 and total RNA concentration >200 ng/μL.

#### 2.6.3 Sample preparation for microarray analysis

All samples that met the required qualitative and quantitative criteria were diluted to a final concentration of 50 ng/μL. Sample preparation was performed according to the *miRNA Microarray System with the miRNA Complete Labeling and Hyb Kit* protocol (version 4.1, October 2021, Agilent Technologies, Santa Clara, CA, United States). The procedure utilized components from the miRNA Labeling and Hybridization Kit and the microRNA Spike-In Kit (Agilent Technologies).

To ensure data normalization and validate each processing step, spike-in controls were added to the diluted samples. RNA was then labeled with pCp-Cy3 fluorescent dye according to the manufacturer’s instructions. The labeled samples were applied to a gasket slide, which was subsequently assembled with a microarray slide. For this study, the *HD Rat miRNA Microarray slide, Release 21.0; 8* × *15K* (Agilent Technologies) was used.

Hybridization was carried out in a hybridization oven at 55°C and 20 rpm for 20 h. Following hybridization, the microarray slides were carefully washed and scanned at a resolution of 5 µm using the Agilent SureScan Dx Microarray Scanner (Agilent Technologies).

#### 2.6.4 miRNA microarray analysis

TIFF images generated by the Agilent SureScan Dx device were processed and converted into text data files using Feature Extraction Software 12.2.0.7 (Agilent Technologies). The qualitative parameters of each sample were assessed in the *Quality Report*, and only samples meeting all required criteria were included in subsequent analyses.

The extracted data were imported into GeneSpring 15.5 GX software (Agilent Technologies) for miRNA expression analysis. Initially, the data were normalized to the 90th percentile and filtered based on quality control *flags*. In GeneSpring, *flags* refer to attributes that indicate the quality of individual features on the microarray chip, including factors such as signal saturation and uniformity.

The normality of the dataset was assessed using the Shapiro-Wilk test. As the data did not follow a Gaussian distribution, non-parametric the Kruskal–Wallis test was applied to evaluate differences in miRNA expression across the analyzed groups (sampling: control, n = 8; SEA0.3, n = 11; SEA3, n = 10). Results were considered statistically significant if the p-value was <0.05 and the fold-change (FC) exceeded ±2.

### 2.7 Gene`s promoter methylation status evaluation

#### 2.7.1 DNA extraction and bisulfite conversion

The mechanical disruptor TissueLyser LT (Qiagen, Germany) was utilized to homogenize and disrupt fresh frozen tissue, designed specifically for low-to-medium disruption of various tissue types. Initially, a tissue sample (∼100 mg) and a 5 mm stainless steel bead were placed into a precooled 2 mL round-bottom tube. The animal tissue was then homogenized in 200 mL lysis buffer at 50 Hz for 1 min. Then, 20 proteinase K was added for sample digestion and incubated at 56°C overnight. Purification of total DNA from animal tissue was carried out using a DNeasy blood and tissue kit (Qiagen, Germany) as per the manufacturer’s protocol. The concentration of the samples was determined using a Qubit™ 3.0 fluorometer (Thermo Fisher Scientific) with a Qubit dsDNA BR assay kit (Thermo Fisher Scientific). Subsequently, isolated DNA (at a concentration of at least 50 ng/μL) was bisulfite-treated using an EpiTect bisulfite kit (Qiagen, Germany) following the manufacturer’s protocol.

#### 2.7.2 CpG assays and determination of methylation status by pyrosequencing

We conducted pyrosequencing analysis of specific regions of target genes, including PTEN, TIMP3, RASSF1A, ATM, and PITX2, using commercially available CpG assays (PyroMark CpG Assay, Qiagen, Germany). The primer sequences can be found in the Supplementary Material of the manuscript. Bisulfite-converted DNA served as the template and was amplified using a PyroMark PCR kit (Qiagen, Germany). PCR conditions included an initial denaturation of DNA for 15 min at 95°C, followed by 45 cycles (96°C for 30 s, 56°C for 30 s, and 72°C for 30 s), and a final extension at 72°C for 10 min. Visualization of PCR products was done using gel electrophoresis (1.75% agarose gel). Subsequently, the PCR products were processed according to the manufacturer’s protocols and analyzed by PyroMark Q96 ID System (Qiagen, Germany) and PyroMark Gold Reagents (Qiagen, Germany). The methylation level of selected CpG dinucleotides was automatically calculated using PyroMark Q96 software version 2.5.8 (Qiagen, Germany).

### 2.8 Serum cytokine levels

Blood samples from mice were centrifuged at 2,000× g for 10 min to form clots. Subsequently, serum samples were obtained and assessed for IL-6, IL-10, TNF-α, and TGF-β cytokines. The levels of these cytokines were measured using ELISA *in vitro* kits from Abcam, Cambridge, MA, United States, specifically designed for quantitative analysis of cytokines in mouse serum (Mouse IL-6 *in vitro* ELISA Kit (ab234570), Mouse IL-10 *in vitro* ELISA Kit (ab214566), Mouse TNF-α *in vitro* ELISA Kit (ab236712), and Mouse TGF-β *in vitro* ELISA Kit (ab119558).

### 2.9 Cell lines, cell cultures, and experimental design

The BC cell lines MCF-7 (HTB-22™, human breast adenocarcinoma) and MDA-MB-231 (HTB-26™, human triple-negative BC) were cultured in high-glucose Dulbecco’s Modified Eagle’s Medium (DMEM; Biosera, Kansas City, MO, United States), supplemented with 10% fetal bovine serum (FBS; Biosera, Kansas City, MO, United States) and 1% antibiotic/antimycotic solution (Merck, Darmstadt, Germany).

Non-cancerous MCF-10A cells (CRL-10317™, human mammary gland epithelial cells) were maintained in high-glucose DMEM/F12 medium supplemented with insulin, epidermal growth factor (EGF), hydrocortisone (HC) (Merck, Darmstadt, Germany), and 10% FBS (Gibco, Thermo Scientific, Rockford, IL, United States). The BJ-5ta cell line (CRL-4001™, human dermal fibroblasts) was cultured in a medium mixture consisting of high-glucose DMEM: M199 at a 4:1 ratio (Biosera, Kansas City, MO, United States), supplemented with Hygromycin B (0.01 mg/mL; Merck, Darmstadt, Germany) and 10% FBS (Gibco, Thermo Scientific, Rockford, IL, United States).

All cell cultures were maintained at 37°C in a humidified incubator with a 5% CO_2_ atmosphere.

### 2.10 Cytotoxicity assay

A resazurin reduction-based assay was employed to evaluate the effects of various *H. rhamnoides* extracts (aqueous, ethanolic, methanolic, and hexane) and cisplatin on cell metabolic activity. The BC cell lines MCF-7 and MDA-MB-231 were seeded in 96-well culture plates at a density of 5 × 10^3^ cells per well and maintained under standard culture conditions. As non-cancerous *in vitro* models, the BJ-5ta and MCF-10A cell lines were used.

After 24 h of incubation, cells were treated with *H. rhamnoides* extracts at concentrations ranging from 500 to 2,000 μg/mL and cisplatin at concentrations ranging from 10 to 100 µM. Cells were further incubated for 72 h. Subsequently, 10 µL of resazurin dye was added to each well, followed by an additional 1.5-h incubation. Fluorescence intensity was measured using the automated Cytation™ 3 Cell Imaging Multi-Mode Reader (Biotek, Winooski, VT, United States). The half-maximal inhibitory concentration (IC_50_) values were determined using the predictive TREND function. DMSO was tested at the highest v/v% concentration corresponding to 2,000 μg/mL of *H. rhamnoides* extracts to assess its potential effects.

To evaluate the potential genoprotective properties of the ethanolic extract (SEAEtOH) in combination with cisplatin, IC_12_._5_ and IC_25_ concentrations of SEAEtOH were tested alongside the IC_50_ concentration of cisplatin in MCF-7 and MDA-MB-231 cells. SEAEtOH was applied as a 1-h pre-treatment before cisplatin administration. Treated samples were incubated for 72 h and analyzed as described above.

### 2.11 Statistical analyses

Data obtained from rodent studies were presented as mean ± SD. Statistical methods such as one-way analysis of variance (ANOVA), Kruskal–Wallis test, Student’s t-test, and Mann–Whitney test were utilized for data analysis. Tumor volumes were calculated using the formula: *V* = π × (S1)2 × S2/12 (where S1 and S2 represent tumor diameters with S1 being less than S2). On the other hand, data from *in vitro* studies were displayed as mean ± SD and were subjected to the ANOVA test followed by the Bonferroni multiple comparisons test. Statistical significance was considered at p ≤ 0.05. The data analyses were conducted using GraphPad Prism Comparison Software (version 5.01, La Jolla, CA, United States).

## 3 Results

### 3.1 Evaluation of secondary metabolites in *Hippophae rhamnoides* fruit peel extract

The content of catechine was determined to be 0.07048 mg/g of *H. rhamnoides* fruit peel. Furthermore, the analysis showed the presence of glycosides derived from quercetin (0.639 mg/g of fruit peel), kaempferol glycosides (0.137 mg/g of fruit peel), and isorhamnetin (3.216 mg/g of fruit peel). The sum of flavonoid glycosides after hydrolysis was established to 3.992 mg/g, re-calculated according to the Pharmacopeia (Ph. Eur. 11.0) was 5.231 mg/g of fruit peel, respectively. The carotenoid analysis showed the presence of 0.0217 mg of lutein per g of the fruit peel and 0.0546 mg of zeaxanthin per g of the *H. rhamnoides* fruit peel. The total amount of carotenoids calculated as lutein and zeaxanthin was 0.0763 mg per g of the fruit peel (Table 1).

**TABLE 1:** Quantification of main secondary metabolites detected in *Hippophae rhamnoides* fruit peel.

Compound	Concentration in the fruit peel (mg/g)	Error of quantification (rel. %)
Isorhamnetin glycosides	3.216 (3.133)	0.5
Quercetin glycosides	0.639 (0.596)	1.0
Kaempferol glycosides	0.137 (0.120)	10.0
Catechine	0.07048	1.0
Zeaxanthin	0.0546	5.0
Lutein	0.0217	5.0

We analyzed and determined the content of carotenoids in n-hexane extract and catechine and flavonoids in EtOH: H2O 2:1 (v/v) extract. For catechine and flavonoids, we utilized LC-DAD-MS, for carotenoids LC-DAD, analysis, respectively. Flavonoid and their glycosides were calculated according to Ph. Eur. 11.0 as hypothetic glycoside.

### 3.2 Therapeutic mouse 4T1 model

#### 3.2.1 Tumor growth and histopathology

**FIGURE 1:**
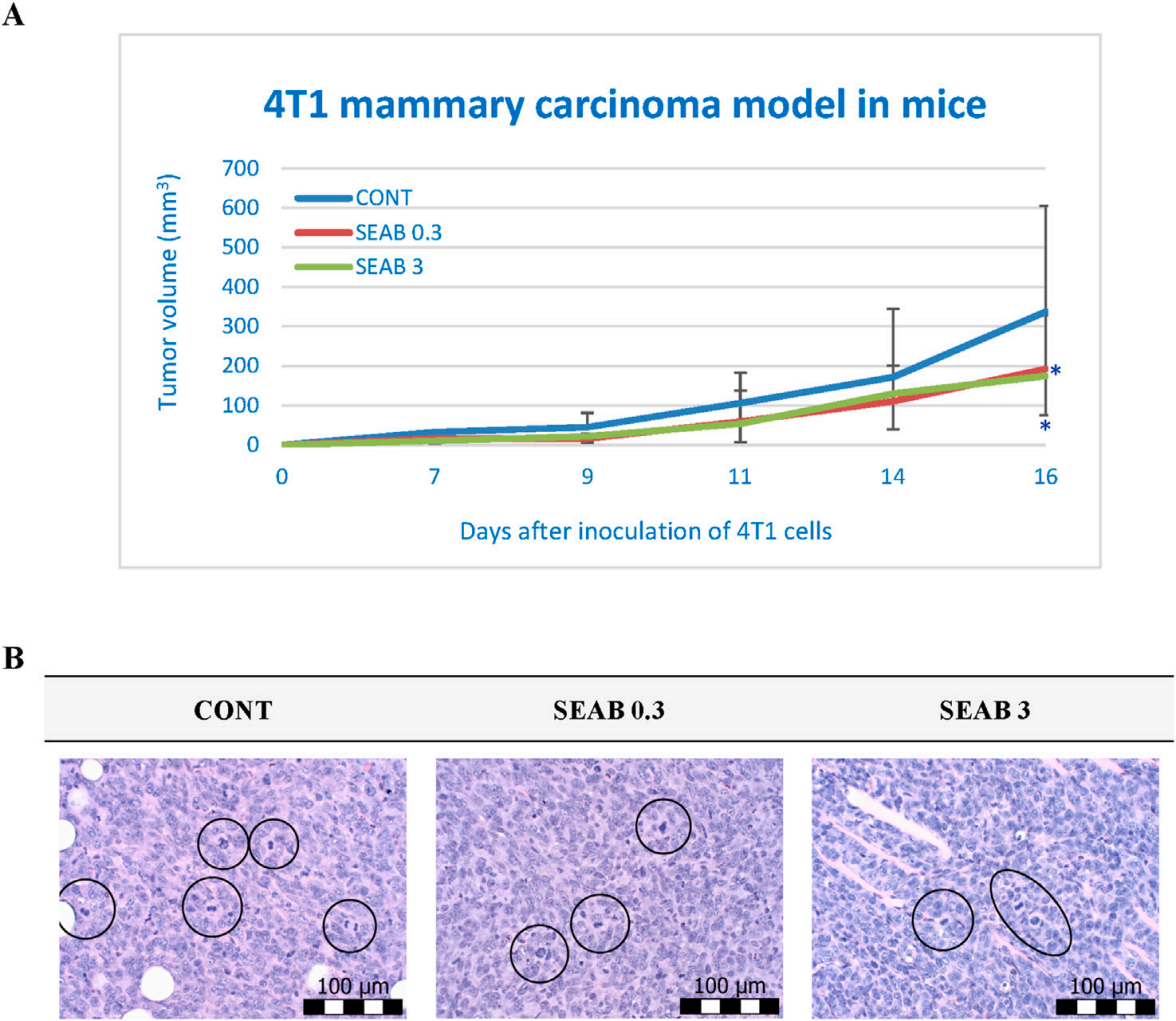
Allograft 4T1 model in mice. **(A)** The progression of 4T1 mammary adenocarcinoma volume in a mouse allograft model following *Hippophae rhamnoides* L. treatment is shown. Data are presented as mean ± SD, with statistical significance indicated at *P < 0.05 compared to the control group (CONT). On day 16, the 95% confidence intervals (CIs) for mean tumor volumes were: CONT [203.31, 470.55], SEAB0.3 [126.85, 258.17], and SEAB3 [128.24, 221.46]. **(B)** The mitotic activity index in 4T1 tumors of Balb/c mice after treatment with *H. rhamnoides* extract is illustrated. Mitotic figures are encircled for emphasis; sections were stained with H&E and observed at a magnification of ×400. CONT represents the untreated control group; SEAB 0.3 denotes the group receiving seaberry at a dietary concentration of 3 g/kg, and SEAB 3 represents the group administered seaberry at 30 g/kg in the diet.

Both doses of seaberry exhibited significant efficacy in reducing the volume of 4T1 tumors in mice at the end of the study. Relative to the untreated control group (CONT), seaberry treatment decreased tumor volume in a dose-dependent manner by 43% (P < 0.05) in the low-dose group and 48% (P < 0.05) in the high-dose group (Figure 1).

Histopathological analysis demonstrated a significant reduction in the mitotic index of cancer cells, with decreases of 34% (P < 0.001) in the low-dose group and 44.5% (P < 0.001) in the high-dose group compared to the control group (CONT) (Table 2; Figure 1).

**TABLE 2:** Histopathological characteristics of 4T1 tumors in Balb/c mice after *Hippophae rhamnoides* treatment.

Parameter	CONT	SEAB 0.3	SEAB 3
Necrosis/whole tumor area	0.028 ± 0.075 [−0.0093, 0.065]	0.016 ± 0.024 [0.0044, 0.028]	0.027 ± 0.034 [0.011, 0.043]
Mitotic activity index	55.06 ± 9.82 [50.46, 59.66]	36.42 ± 8.23*** [32.45, 40.39]	30.65 ± 8.39***+ [26.72, 34,58]

Data are expressed as mean ± SD. A significant difference, ***P < 0.001 vs CONT, + P < 0.05 vs. SEAB, 0.3. A 95% confidence interval (CI) of the mean is expressed in square brackets.

However, no significant differences were observed in the necrosis-to-tumor area ratio among the experimental groups.

#### 3.2.2 Serum cytokine levels

**FIGURE 2:**
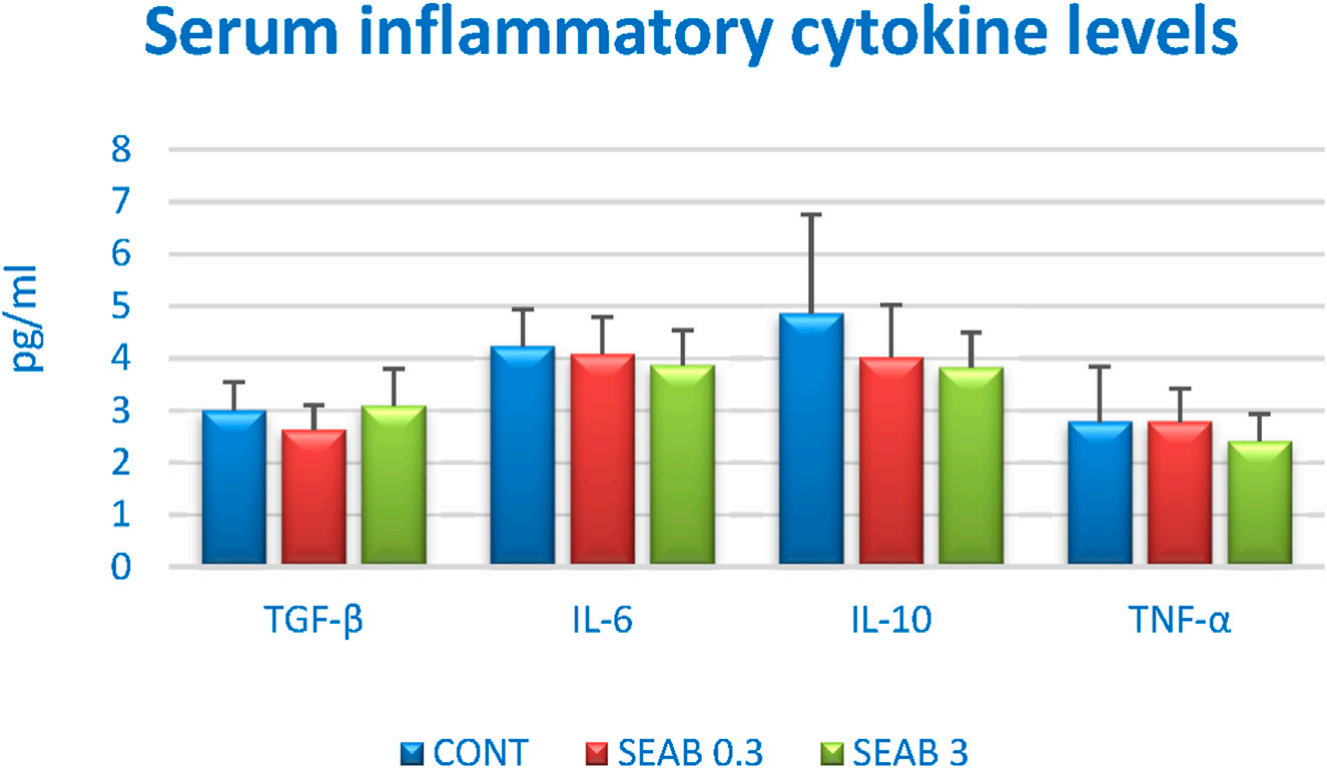
Serum inflammatory cytokine levels in 4T1 mouse model after *Hippophae rhamnoides* treatment. Data are shown as mean ± SD.

The serum inflammatory cytokine levels in the mouse 4T1 BC model are summarized in Figure 2. While no statistically significant differences were observed among the experimental groups, a dose-dependent decrease in serum levels of IL-6, IL-10, and TNF-α was evident following seaberry treatment, except for TGF-β. Notably, the reduction in IL-10 levels approached statistical significance when comparing the SEAB3 group to the control group (CONT) (P = 0.06).

### 3.3 Chemoprevention of rat mammary carcinogenesis

The chemopreventive effects of seaberry in an experimental rat model of BC demonstrated a significant reduction in the ratio of poorly to well-differentiated carcinomas (high-grade/low-grade, HG/LG). Specifically, the higher seaberry dose reduced the HG/LG ratio by 58.5% (P = 0.029) compared to carcinomas in the control group (Table 3). In contrast, low-dose seaberry administration resulted in a non-significant 43.5% reduction in the HG/LG ratio (P = 0.16) relative to the control.

**TABLE 3:** Effects of *Hippophae rhamnoides* administration in chemically induced rat mammary carcinogenesis at the end of the experiment.

Group	CONT	SEAB 0.3	SEAB 3
Tumor-bearing animals/all animals	19/25	17/24	20/25
Tumor incidence (%)	76.00	70.08	80.00
Tumor frequency per group*	2.12 ± 1.86 [1.35, 2.89]	2.13 ± 1.92 [1.32, 2.94]	2.24 ± 2.54 [1.19, 3.29]
Tumor latency* (days)	73.68 ± 11.52 [68.13, 79.23]	74.94 ± 10.13 [69.73, 80.15]	74.20 ± 17.50 [66.01, 82.39]
Average tumor volume* (cm^3^)	0.594 ± 0.809 [0.38, 0.81]	0.563 ± 0.743 [0.36, 0.77]	0.542 ± 0.659 [0.37, 0.72]
High/low-grade carcinomas ratio	26/27 (=0.963)	18/33 (0.545)	16/40 * (0.400)

CONT, control group; SEAB, 0.3 – a group with administered seaberry at a concentration of 3 g/kg in the diet, SEAB, 3 – a group with administered seaberry at a concentration of 30 g/kg in the diet. Data are expressed as means ± SD. Significantly different, * P < 0.05 vs CONT. A 95% confidence interval (CI) of the mean is expressed in square brackets.

However, seaberry treatment did not exhibit any significant effects on other parameters of rat mammary carcinogenesis, including tumor frequency, latency, incidence, and volume.

Histopathological examination of mammary tumor samples identified mixed papillary/cribriform, cribriform/papillary, and single cribriform carcinomas as the most prevalent mammary lesions, with the first type being dominant. Less frequently observed lesions included mixed cribriform/comedose, cribriform/papillary/comedose, and tubular/cribriform carcinomas.

### 3.4 Immunohistochemistry of rat tumors

#### 3.4.1 Markers of apoptosis, proliferation, and angiogenesis

Seaberry treatment demonstrated a dose-dependent increase in two apoptotic markers: cytoplasmic caspase-3 and Bax expression. Cleaved caspase-3 expression was upregulated by 35.5% in the SEAB 0.3 group (P = 0.076) and 61% in the SEAB 3 group (P < 0.01) compared to control tumor samples. Bax protein levels increased by 15% (P = 0.18) in the low-dose group and 31.5% (P < 0.01) in the high-dose group relative to controls.

The expression of the anti-apoptotic protein Bcl-2 was reduced by approximately 20% in both seaberry-treated groups, although this change was not statistically significant. Importantly, the Bax/Bcl-2 ratio was significantly elevated in the treated groups, increasing by 78.5% in the SEAB 0.3 group (P < 0.05) and 74.5% in the SEAB 3 group (P < 0.01) compared to controls.

Seaberry treatment at higher doses reduced the expression of the proliferation marker Ki67 by 42%, while the angiogenesis marker VEGF was reduced dose-independently by 28% (P < 0.001) and 22.5% (P < 0.001) in the SEAB 0.3 and SEAB 3 groups, respectively, compared to controls.

**FIGURE 3:**
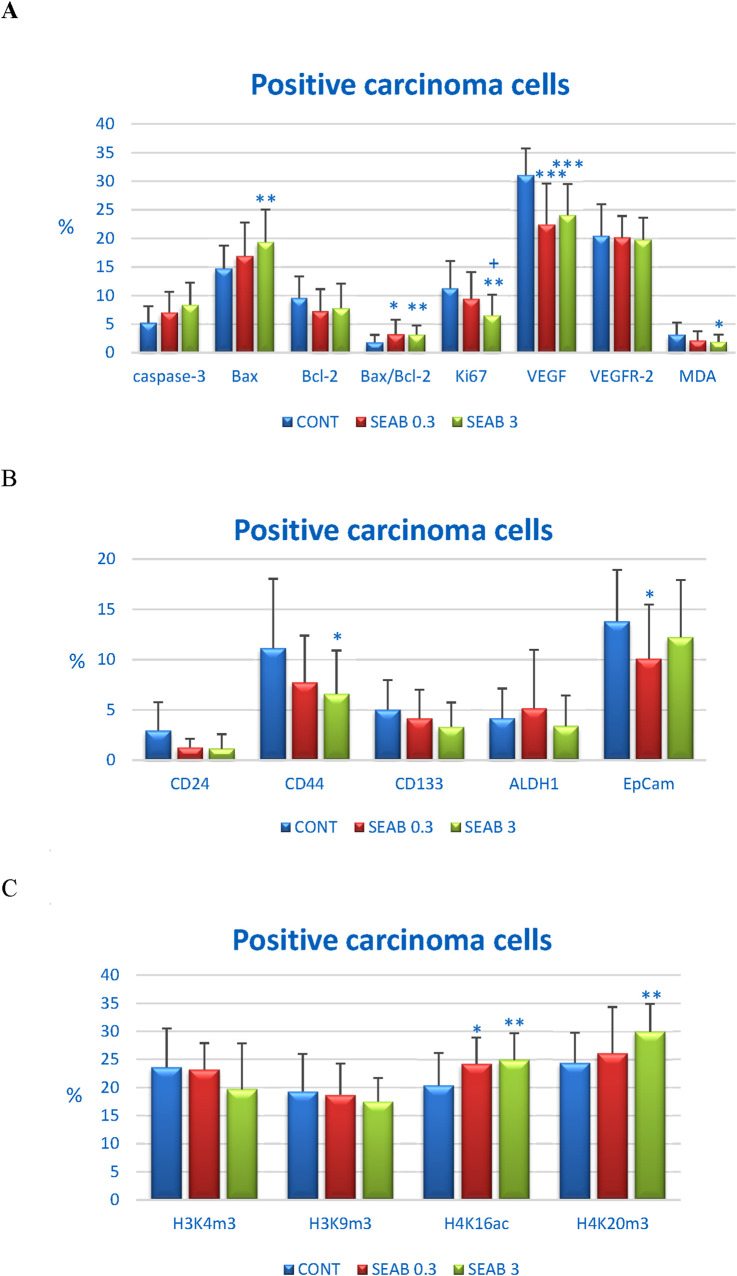
Immunohistochemical analysis of rat carcinoma cells following *Hippophae rhamnoides* treatment. **(A)** Immunoexpression levels of cleaved caspase-3 (cytoplasmic), Bax, Bcl-2, Ki67, VEGFA, VEGFR-2, and MDA in rat tumor samples. **(B)** Immunoexpression levels of cancer stem cell markers in rat tumor samples. **(C)** Immunoexpression levels of histone markers H3K4m3, H3K9m3, H4K16ac, and H4K20m3 in rat tumor samples. Data are presented as mean ± SD. Statistical significance is indicated as *P < 0.05, **P < 0.01, ***P < 0.001 *versus* the control group (CONT), and +P < 0.05 *versus* the SEAB 0.3 group. Protein expression is quantified as the mean percentage of antigen-positive area in standardized fields (0.5655 mm^2^) within hotspot regions of the tumor area. A minimum of 60 images were analyzed for each parameter.

Additionally, seaberry decreased cytoplasmic levels of malondialdehyde (MDA), a marker of lipid oxidation, in a dose-dependent manner by 32% (P = 0.081) and 39.5% (P < 0.05) relative to the control group. VEGFR-2 expression, however, remained unaffected by seaberry treatment (Figure 3A).

#### 3.4.2 Markers of cancer stem cells

The *in vivo* analysis of cancer stem cell (CSC) markers revealed a noticeable but borderline significant reduction in CD24 expression in both seaberry-treated groups. CD24 expression decreased by 57% in the SEA 0.3 group (P = 0.07) and by 61.5% in the SEA 3 group (P = 0.07) compared to control tumor samples.

Seaberry treatment also reduced CD44 expression by 30.5% (P = 0.08) at the lower dose and by 41% (P = 0.02) at the higher dose relative to controls. CD133 expression was borderline significantly downregulated by 33.5% (P = 0.06) following high dose seaberry treatment compared to the control group.

No significant changes were observed in the expression levels of ALDH1 and EpCAM, other CSC markers, between treated and control tumor samples (Figure 3B).

#### 3.4.3 Markers of histone chemical modification

The post-translational chemical modifications of histones H3 and H4 in carcinoma cells from an *in vivo* rat model following seaberry administration are summarized in Figure 3C. Seaberry treatment dose-dependently upregulated H4K16ac levels by 19% (P < 0.05) and 22.5% (P < 0.01) compared to control samples. Additionally, high dose seaberry increased H4K20m3 levels by 23% (P < 0.01) relative to control carcinomas.

A mild, dose-dependent, yet statistically non-significant reduction in H3K4m3 levels (16.5% decrease in SEAB3 compared to CONT) and H3K9m3 levels was observed following seaberry treatment (Figure 3C).

**FIGURE 4:**
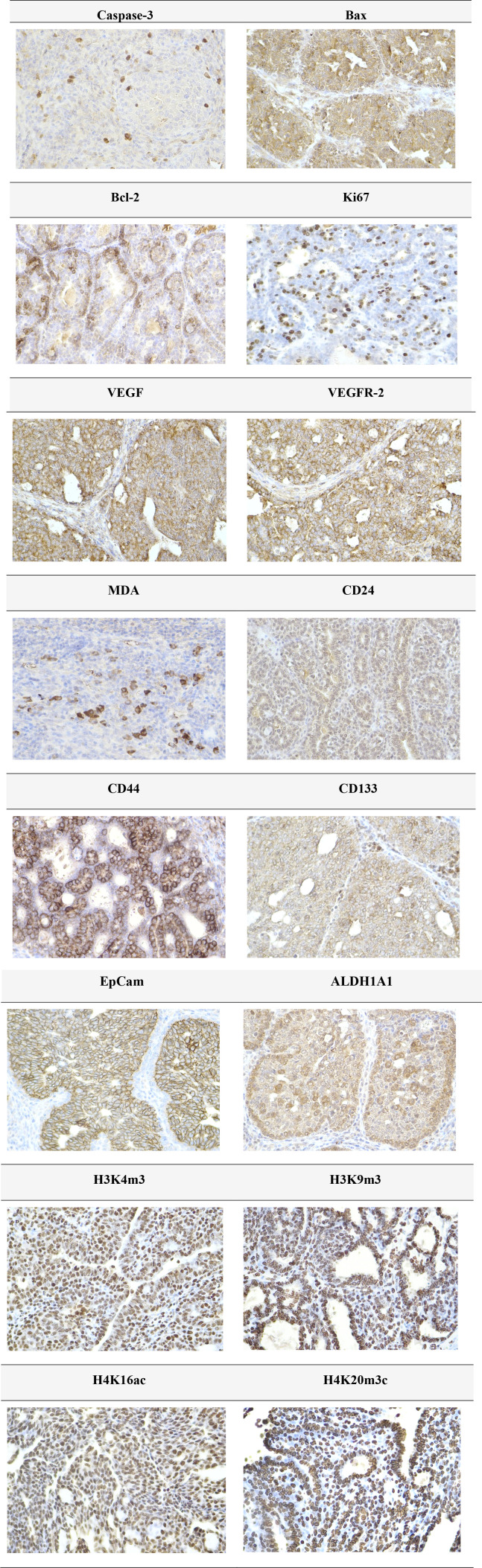
Representative immunohistochemical images illustrating the expression of cleaved caspase-3, Bax, Bcl-2, Ki67, VEGFA, VEGFR-2, MDA, CD24, CD44, CD133, ALDH1A1, EpCAM, H3K4m3, H3K9m3, H4K20m3, and H4K16ac in carcinoma tissue of the rat mammary gland. For detection, the following antibodies were used: polyclonal caspase-3 antibody (Bioss, Woburn, United States), polyclonal Bax and Bcl-2 antibodies (Santa Cruz Biotechnology, Paso Robles, CA, United States), monoclonal Ki67 antibody (Dako, Glostrup, Denmark), monoclonal VEGFA and VEGFR-2 antibodies (Santa Cruz Biotechnology, Paso Robles, CA, United States), polyclonal CD24 antibody (GeneTex, Irvine, CA, United States), polyclonal CD44 antibody (Boster, Pleasanton, CA, United States), polyclonal ALDH1A1 antibody (ThermoFisher, Rockford, IL, United States), and polyclonal MDA, EpCAM, H3K4m3, H3K9m3, and H4K20m3 antibodies, along with monoclonal H4K16ac antibody (Abcam, Cambridge, MA, United States). Images were captured at a final magnification of ×400.

The expression patterns of cleaved caspase-3, Bax, Bcl-2, Ki67, VEGFA, VEGFR-2, CD24, CD44, CD133, ALDH1A1, EpCAM, H3K4m3, H3K9m3, H4K16ac, and H4K20m3 in rat BC samples are depicted in Figure 4.

### 3.5 Assessment of differences in MiRNA expression in tumour samples *In vivo*

Using the microarray approach on the Agilent SureScan DX platform, we quantified the expression levels of 758 miRNAs across 29 individual samples. Statistical analysis of differential expression was conducted using the Kruskal–Wallis test. Our findings revealed significant alterations in the expression of nine miRNAs following the administration of seaberry at dietary concentrations of 0.3% or 3%, compared to the control group. A comprehensive summary of all upregulated and downregulated miRNAs, along with their corresponding p-values and fold-change (FC) values, is presented in Table 4.

**TABLE 4:** Differentially expressed miRNAs with statistically significant variation among the analyzed groups.

	p (CONT vs. SEA0.3 vs. SEA3)	FC (CONT vs. SEA0.3)	FC (CONT vs. SEA3)	FC (SEA0.3 vs. SEA3)
Upregulated				
rno-miR-10a-5p	0.0070	1.68	**2.06**	−1.23
rno-miR-322-5p	0.0003	**2.54**	1.72	1.48
rno-miR-450a-5p	0.0041	**6.85**	1.47	**4.67**
Downregulated				
rno-miR-142-5p	0.0127	**−2.07**	**−2.55**	1.23
rno-miR-148b-3p	0.0334	1.38	**−2.30**	**3.17**
rno-miR-1839-3p	0.0276	**−2.12**	**−2.01**	−1.06
rno-miR-18a-5p	0.0075	1.78	**−2.13**	**3.80**
rno-miR-1949	0.0236	−1.69	**−2.26**	1.34
rno-miR-347	0.0200	**−2.09**	**−3.35**	1.60

CONT, represents the untreated control group; SEAB, 0.3 denotes the group receiving seaberry at a dietary concentration of 3 g/kg, and SEAB, 3 represents the group administered seaberry at 30 g/kg in the diet. Numbers in bold, **FC** is greater than **2**. FC, fold-change; rno, *rattus norvegicus*.

The administration of seaberry significantly increased the expression of three tumor-suppressive miRNAs in BC tissue. Specifically, miR-10a-5p was upregulated in the SEA3 group, while miR-322-5p and miR-450a-5p exhibited increased expression in the SEA0.3 group (Table 4).

Conversely, six miRNAs were significantly downregulated in tumor tissue following seaberry treatment. Among these, three miRNAs—miR-142-5p (oncogenic), miR-1839-3p, and miR-347—displayed a FC greater than two after administration of both seaberry doses. However, three additional miRNAs—miR-148b-3p (oncogenic), miR-18a-5p (dual role), and miR-1949—were significantly downregulated only in response to the higher seaberry dose (Table 4).

Furthermore, differential expression analysis between treatment groups (SEA0.3 vs SEA3) revealed significant modulation of three miRNAs. In the SEA3 group, tumor-suppressive miR-450a-5p was downregulated (FC = 4.67), while oncogenic miR-148b-3p and miR-18a-5p were upregulated (FC = 3.17 and 3.80, respectively) (Table 4).

### 3.6 Tumor suppressor genes: promoter methylation status *In vivo*

**FIGURE 5:**
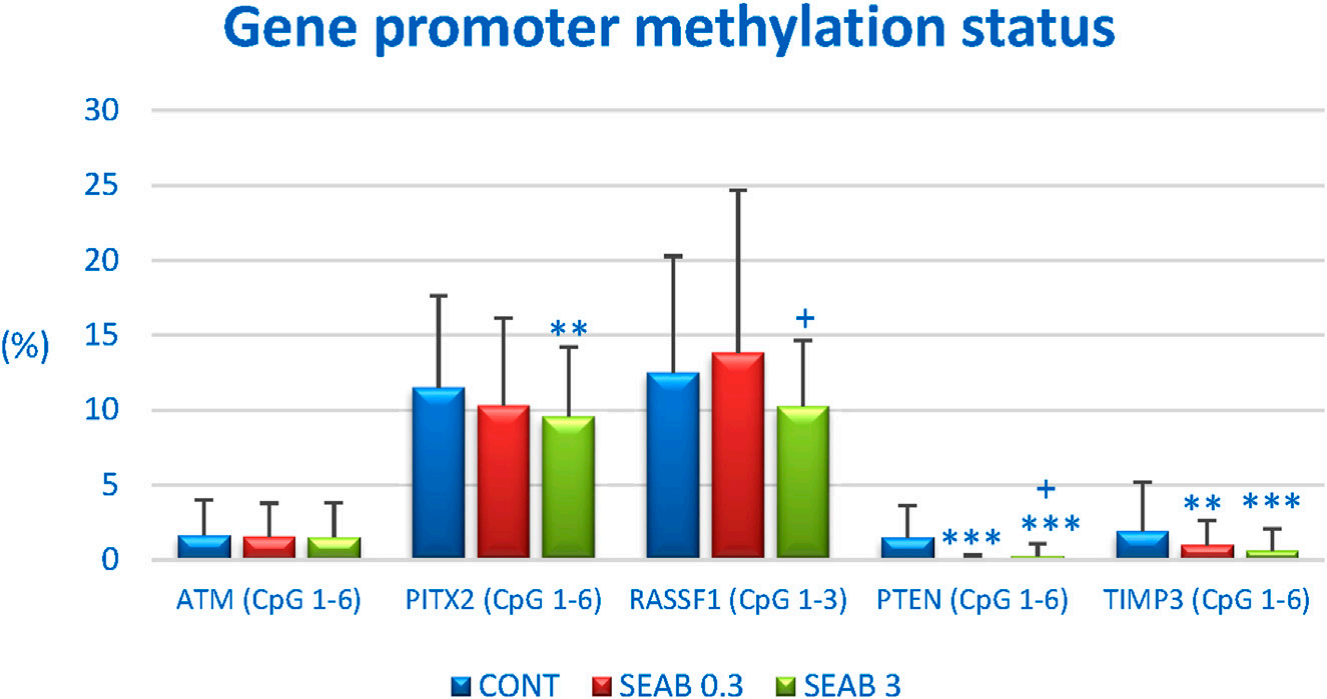
The methylation status of the promoter regions for the tumor-suppressor genes *ATM*, *PITX2*, *RASSF1A*, *PTEN*, and *TIMP3* in rat BC samples. The methylation levels were determined based on the analysis of all evaluated CpG islands within the promoter regions of these genes, with the number of evaluated islands indicated in brackets. Results are presented as mean ± standard deviation (SD). Statistically significant differences are denoted as **p < 0.01 or ***p < 0.001 compared to the CONT group, and ++p < 0.01 compared to the SEAB 0.3 group.

Promoter methylation analysis was conducted for the following tumor-suppressor genes: *ATM* (NPAT) with four analyzed CpG sites (CpG 1–4), *PITX2* (CpG 1–5), *RASSF1* (CpG 1–3), *PTEN* (CpG 1–6), and *TIMP3* (CpG 1–6) (Figure 5). A total of 20 BC specimens per experimental group were analyzed as part of a rat chemoprevention study.

Seaberry treatment significantly reduced the overall methylation of the *PTEN* promoter by 96% (p < 0.001) at lower doses and by 85.5% (p < 0.001) at higher doses compared to control samples. For the *TIMP3* promoter, a dose-dependent decrease in methylation of 49% (p < 0.01) and 68.5% (p < 0.001) was observed following seaberry treatment, relative to untreated tumor samples. Methylation analysis of the *ATM (NPAT)* promoter revealed a dose-dependent but non-significant reduction of 6.5% and 7.5% compared to control samples. Additionally, higher doses of seaberry resulted in a 17% reduction in *PITX2* promoter methylation (p < 0.01) and an 18% reduction in *RASSF1* promoter methylation (p = 0.054) compared to controls (Figure 5).

### 3.7 Physiological *in vivo* effects

At the end of both rodent studies, seaberry administration did not significantly change weight gain or food intake. Continuous dietary supplementation with seaberry over 14 weeks was well tolerated in rats. No macroscopic organ abnormalities, such as liver steatosis, hepatic or splenic enlargement, or signs of gastritis, were observed during the autopsy. Additionally, no hematopoietic disorders or other adverse effects were detected, including abnormalities in hair, mucosa, or vitality.

The average daily seaberry dose per rat was 50.7 mg in the SEA 0.3 group and 474.0 mg in the SEA 3 group. In mice, the average daily doses were 9.84 mg (SEA 0.3) and 103.5 mg (SEA 3).

### 3.8 *In vitro* analyses–screening

To assess potential inhibitory effects, four different extracts of *H. rhamnoides* were tested on the BC cell lines MCF-7 and MDA-MB-231, as well as two non-cancerous cell lines (MCF-10A and BJ-5ta). The predicted IC_50_ values are presented in Table 5.

**TABLE 5:** Calculated IC_50_ for various *Hippophae rhamnoides* extracts against BC cell lines and non-cancer cells.

	SEA_H2O_ (µg/mL)	SEA_HEX_ (µg/mL)	SEA_MeOH_ (µg/mL)	SEA_EtOH_ (µg/mL)	CisPt (µM)	DMSO v/v 5%
MCF-7	3,344 ± 874 [1,173, 5,515]	2,459 ± 377 [1,522, 3,396]	2,104 ± 306 [1,344, 2,864]	1,902 ± 332 [1,077, 2,727]	29.7 ± 1.6 [25.7, 33.7]	2,536 ± 509 [1,272, 3,800]
MDA-MB-231	2,306 ± 35 [2,219, 2,393]	2,006 ± 658 [371, 3,641]	1,564 ± 333 [737, 2,391]	1,155 ± 174 [723, 1,587]	7.1 ± 2.2 [1.6, 12.6]	2,058 ± 432 [985, 3,131]
MCF-10A	1,853 ± 343 [1,001, 2,705]	1,732 ± 337 [895, 2,569]	1,669 ± 337 [832, 2,506]	1,059 ± 96 [821, 1,297]	25.9 ± 2.6 [19.4, 32.4]	1,312 ± 304 [557, 2,067]
BJ-5ta	1,319 ± 383 [368, 2,270]	1,323 ± 17 [1,281, 1,365]	1,356 ± 202 [854, 1,858]	1,028 ± 373 [101, 1,955]	37.9 ± 2.9 [30.7, 45.1]	865 ± 63 [709, 1,022]

^a^ Results are presented as mean (SD) from three independent experiments. A 5% v/v DMSO solution corresponds to 2 mg/mL. Ninety-five percent confidence intervals (CIs) for the mean are indicated in square brackets.

Among the four extracts, the ethanolic extract exhibited the strongest inhibitory effects on both cancer cell lines, followed by the methanolic extract. In contrast, the aqueous extract demonstrated the weakest inhibitory activity, with the highest predicted IC_50_ values.

To evaluate the potential contribution of the solvent, DMSO at a higher concentration (5% v/v, equivalent to 2 mg/mL) was also tested. The predicted IC_50_ values for DMSO are shown in Table 5. The results indicate that at higher concentrations, DMSO may influence the inhibitory effects of the tested extracts.

For subsequent experiments, the IC_50_ value of cisplatin was also determined.

### 3.9 *In vitro* analyses–protective effect of the ethanolic extract

**FIGURE 6:**
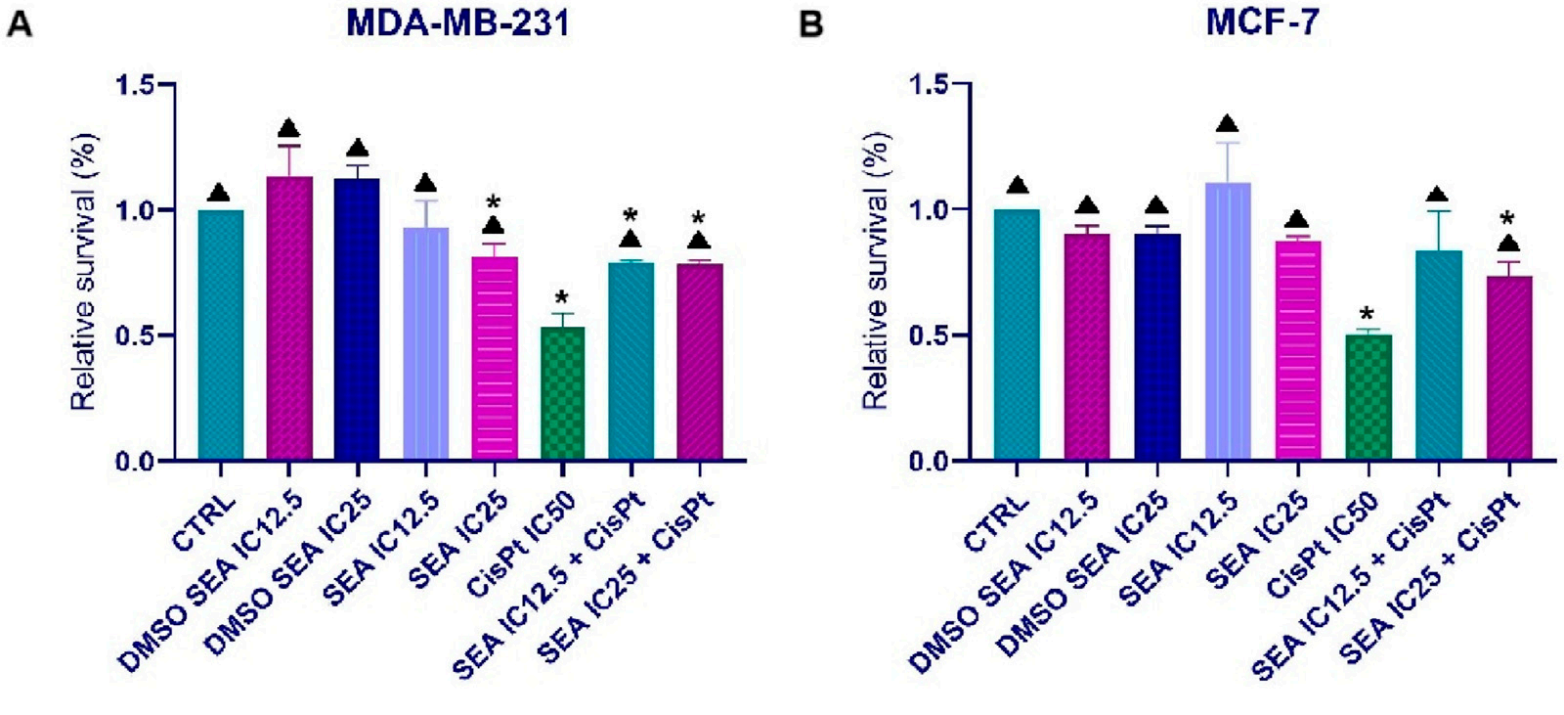
Effects of SEAEtOH and cisplatin combinations on the metabolic activity of MDA-MB-231 **(A)** and MCF-7 **(B)** cells. Relative survival values are presented as the mean ± standard deviation from three independent experiments. (*p < 0.05 compared to the control; ▲p < 0.05 compared to the IC_50_ of cisplatin, as determined by ordinary one-way ANOVA with Dunnett’s *post hoc* test).

The potential protective effect of the ethanolic extract was evaluated in both BC cell lines (Figure 6). For this purpose, two low concentrations (IC_12_._5_ and IC_25_), prepared by serial dilution of the IC_50_ value, were used as a 1-h pre-treatment before cisplatin exposure.

As shown in Figure 6, pre-treatment with the SEAEtOH extract partially protected MCF-7 and MDA-MB-231 BC cells from the cytotoxic effects of cisplatin (IC_50_). These findings suggest a potential genoprotective role of ethanolic extract.

## 4 Discussion

Findings from phase I, II, and III clinical trials suggest that selected phytochemicals and whole plant-based foods (Yardley et al., 2010; Crew et al., 2012; Khan et al., 2012; Samavat et al., 2015; Cortes et al., 2018; Campbell et al., 2024), characterized by low toxicity during long-term administration in humans, may provide a gradual yet effective therapeutic approach for cancer patients or individuals at high risk of developing cancer. To identify novel plant-derived compounds with potent anticancer properties for potential inclusion in future therapeutic or preventive strategies, we investigated the oncostatic effects of *H. rhamnoides* (seaberry) in BC (BC) animal models and human cell lines.

The therapeutic dosage of plant-based treatments, including whole foods, varies substantially across mammalian species. Thus, only carefully controlled clinical studies can establish optimal dosing regimens for high-risk individuals or patients. The dietary inclusion of seaberry at 0.3% w/w in the SAE 0.3 group was designed to reflect traditional human consumption patterns, approximating a daily intake of 20 g of dried fruit (Sea Buckthorn Uses, Benefits and Dosage, n. d.). Acknowledging potential interspecies variations in the pharmacokinetics and pharmacodynamics of seaberry phytochemicals, particularly between humans and rodents, we also implemented a tenfold higher dosage (3% w/w) in our rodent studies. This approach aimed to account for metabolic differences and ensure a comprehensive evaluation of seaberry’s effects across species. Moreover, the seaberry doses used in this study were based on previous experience applying various fruit peels in the mouse syngeneic and chemically-induced BC rodent models (Kubatka et al., 2016a; 2020b; Dvorska et al., 2024). The findings of this investigation, along with prior studies, confirmed the suitability of the dosages applied. The secondary metabolites identified in seaberry (*H. rhamnoides*), including isorhamnetin, quercetin-derived glycosides, kaempferol glycosides, catechin, zeaxanthin, and lutein (Table 1), have demonstrated significant anticancer activity against various types of BC in oncology research (Thomson et al., 2007; Duo et al., 2012; Hu et al., 2015; Xiang et al., 2016; Nguyen et al., 2017; Gong et al., 2018; Ma et al., 2023; Maugeri et al., 2023; Yang et al., 2023; Dehnavi et al., 2024). The relevance of seaberry’s phytochemical profile to BC management lies in the synergistic effects of these compounds (Kapinova et al., 2017a). Isorhamnetin has been shown to suppress proliferation and induce apoptosis in BC cells by modulating key signaling pathways, including AMPK/mTOR/p70S6K (Yang et al., 2023), Akt, and MEK1/2 (Hu et al., 2015). Quercetin inhibits BC cell growth and induces apoptosis through mechanisms involving FasL, p53, p21, GADD45 (Nguyen et al., 2017), Bcl-2, and Bax (Duo et al., 2012). Catechins induce apoptosis and inhibit proliferation by arresting the cell cycle, promoting TP53/caspase-mediated pathways, downregulating anti-apoptotic proteins, inhibiting fatty acid synthase, and modulating the nitric oxide synthase system (Xiang et al., 2016). The presence of carotenoids like zeaxanthin and lutein further enhances seaberry’s potential, as these compounds possess antioxidant properties that may protect cells from oxidative damage and reduce cancer risk (Thomson et al., 2007; Dehnavi et al., 2024). Lutein, in particular, has been shown to promote growth inhibition of BC cells through increased reactive oxygen species generation and modulation of several signaling pathways (Gong et al., 2018).

In this study, seaberry demonstrated limited chemopreventive efficacy in rats, as evidenced by a significant improvement in the histopathological characteristics of mammary carcinoma lesions, specifically a reduced high-grade/low-grade (HG/LG) carcinoma ratio in the high-dose treatment group. These results align with our previous extensive research using the same chemically induced rat mammary carcinogenesis model. In those studies, interventions with chlorella and a mixture of dark fruit peels, oregano, cloves, thyme, cinnamon, sumac, salvia, and aronia significantly reduced tumor incidence, frequency, and volume, extended tumor latency, and lowered the HG/LG carcinoma ratio (Kubatka et al., 2015; 2016a; 2017a; 2017b; 2019; 2020a; 2020b; 2024; Dvorska et al., 2024). The chemopreventive effects of plant-based foods on mammary carcinogenesis *in vivo* have been corroborated by other researchers. For example, blueberries and blackberries exhibited both preventive and therapeutic properties by decreasing tumor volume and proliferation while prolonging tumor latency (Ravoori et al., 2012; Jeyabalan et al., 2014). Similarly, rosemary significantly reduced tumor frequency in a DMBA-induced rat mammary carcinogenesis model, showcasing pronounced chemopreventive potential (Singletary et al., 1996). Pomegranate has also demonstrated chemopreventive efficacy in carcinogen-induced rat mammary tumors, primarily through pro-apoptotic and antiproliferative mechanisms (Bishayee et al., 2016).

In the triple-negative 4T1 breast adenocarcinoma mouse model, seaberry exhibited significant therapeutic efficacy, evidenced by a marked reduction in tumor volume and the mitotic activity index of carcinoma cells in both treatment groups. Similarly, in our recent study using the same model, aronia demonstrated a dose-dependent reduction in tumor volume and mitotic activity index at both dietary concentrations (0.3% and 3%) compared to the control group (Dvorska et al., 2024). These findings align with previous research by our team, which showed that *Thymus vulgaris* L. significantly decreased 4T1 tumor volume at both doses, while also lowering the mitotic activity index and reducing the necrosis-to-total tumor tissue ratio (Kubatka et al., 2019). Comparable antitumor effects were observed with *Cinnamomum zeylanicum* L., which dose-dependently reduced 4T1 tumor volume and mitotic activity index (Kubatka et al., 2020a). Additionally, our studies demonstrated significant antitumor efficacy of *Rhus coriaria* L. (Kubatka et al., 2020b) and *Salvia officinalis* L. (Kubatka et al., 2024) in the same 4T1 BC model. Collectively, these results confirm that the antitumor effects of phytochemicals are comparable to those of synthetic drugs tested in the same mouse 4T1 model (Demečková et al., 2017; Solár et al., 2017; Grasselly et al., 2018). Despite these promising findings, further rigorous preclinical and clinical investigations are necessary to evaluate the therapeutic potential of plant-based compounds in human BC. It is important to acknowledge the limitations of the 4T1 model, including its reliance on a single tumor cell line and experimental conditions that do not fully replicate the complexity of clinical settings.

Extensive research suggests that the anticancer potential of natural phytochemical complexes in whole foods surpasses that of isolated plant-derived compounds, a conclusion supported by our recent comprehensive review (Kapinova et al., 2017b). The ability of diverse bioactive molecules in plant “superfoods” to simultaneously target multiple carcinogenesis-related signaling pathways offers a promising strategy for BC management. However, the translation of these preclinical findings into clinical applications poses significant challenges. Critical issues requiring resolution include: (1) achieving sufficient plasma concentrations of phytochemicals or their active metabolites in humans, potentially through advanced delivery systems like nanotechnology; (2) obtaining pharmacokinetic profiles, including absorption and excretion data, for various phytochemicals; (3) determining safe and effective dosing of specific plant-derived compounds; (4) understanding synergistic drug combinations that may reduce reliance on conventional therapies; (5) assessing the effects of phytochemicals on key cell signaling pathways; (6) investigating their potential to re-sensitize chemotherapy- or radiotherapy-resistant cancers; (7) evaluating their influence on cancer cell invasion, metastasis, and recurrence risk; and (8) uncovering patient-specific mechanisms to facilitate a more personalized approach to plant-based cancer therapeutics.

The oncostatic properties of medicinal plant-based foods are well-documented, with evidence attributing their effects to mechanisms such as programmed cell death induction, cell cycle and proliferation regulation, angiogenesis inhibition, and antioxidant activity. These effects are mediated by specific combinations of secondary metabolites present in certain plants (Mazurakova et al., 2022). Apoptosis, a tightly regulated process essential for cellular homeostasis, involves multiple proteins and signaling pathways (Abotaleb et al., 2018; Abadi et al., 2022). Among these, caspases and the Bcl-2 family play central roles. Caspases, a group of cysteine proteases, initiate apoptosis and are typically present in cells as inactive precursors, or procaspases. In the Bcl-2 protein family, under cellular stress conditions, anti-apoptotic members such as Bcl-2 inhibit pro-apoptotic proteins, Bax and Bak, thereby preserving mitochondrial membrane integrity.

Apoptosis can occur *via* two principal pathways: intrinsic and extrinsic (Abotaleb et al., 2018). The intrinsic pathway is regulated by Bcl-2 family proteins, where an increased Bax/Bcl-2 ratio disrupts mitochondrial stability, leading to caspase-3 activation and triggering apoptotic events in cancer cells. Substantial evidence highlights the regulatory effects of phytochemicals on Bax/Bcl-2/caspase-3 signaling in cancer (Pal et al., 2016; Jabeen et al., 2020; Nazeri et al., 2020). In this study, seaberry treatment resulted in a significant increase in the Bax/Bcl-2 ratio in rat mammary tumors at both dose levels, accompanied by elevated expression of cleaved caspase-3 in treated groups. Similar observations were reported in recent chemopreventive studies using rat models, where treatments with dark fruit peel, oregano, clove buds, cinnamon, sumac, salvia, and aronia significantly elevated the Bax/Bcl-2 ratio and caspase-3 expression in mammary carcinoma cells (Kubatka et al., 2016a; 2017a; 2017b; 2020a; 2020b; 2024; Dvorska et al., 2024). These findings suggest that seaberry and other specific plant nutraceuticals may serve as effective apoptosis-inducing plant-based interventions in breast carcinogenesis.

Isolated plant secondary metabolites and their natural mixtures present in medicinal plants exert significant effects on the proliferation of BC cells (Adams et al., 2010; Ouhtit et al., 2013; Alateyah et al., 2023; Banerjee et al., 2023). These phytochemicals act on multiple signaling pathways and mechanisms regulating the cell cycle and proliferation, including PI3K, Nrf2, COX-2, NF-κB, poly-ADP-ribosylation, Plk1, STAT3, Hedgehog, Wnt, and epigenetic modifications (Paul et al., 2024). In this study, immunohistochemical analysis of the nuclear protein Ki67, a recognized marker of cell proliferation, revealed a near-significant, dose-dependent reduction in proliferation within rat tumor samples from both treatment groups compared to controls. Additionally, seaberry treatment at both dose levels significantly decreased the mitotic activity index in 4T1 tumors, further supporting its potential as an antiproliferative agent in BC management.

Multiple signaling pathways, including VEGF, EGF, FGF, and HGF, play pivotal roles in endothelial tube formation and angiogenesis within tumor tissues, contributing to the heterogeneity of blood vessel structures observed in cancer. Targeting these angiogenic pathways has emerged as a promising strategy in cancer therapy, with the VEGF-kinase ligand/receptor signaling pathway being particularly critical for neovascularization (Liu et al., 2023). Phytochemicals have demonstrated the ability to modulate VEGF-promoting factors by interacting with and inhibiting these pathways, thereby suppressing cancer growth (Parveen et al., 2019). In this study, *H. rhamnoides* treatment at both dose levels significantly reduced VEGF expression in rat tumor samples. These findings, consistent with previous research from our laboratory (Kubatka et al., 2016a; 2017a; 2017b; 2019; 2020a; Dvorska et al., 2024), highlight the potential of phytochemicals in anti-angiogenic therapy. However, despite encouraging preclinical results, translating these findings into clinical success remains challenging. Clinical trials often yield less favorable outcomes compared to preclinical studies. To address these limitations, future research should prioritize well-designed, randomized, and placebo-controlled clinical trials to comprehensively evaluate the therapeutic potential of phytochemicals and whole medicinal plants in anti-angiogenic cancer therapy.

Imbalances in cellular redox status can result in oxidative modifications to critical biomolecules such as DNA, proteins, and lipids, processes closely associated with carcinogenesis (Juan et al., 2021). Plant-derived antioxidants are thought to play a vital role in cancer prevention by stabilizing these cellular components and mitigating oxidative damage. Extensive research from our laboratory has demonstrated that whole plant foods or bioactive substances, administered in relatively low dietary doses—including young barley leaves, dark fruit peels, clove buds, thyme, cinnamon bark, sumac, and salvia—can significantly reduce oxidative damage to lipids and proteins in BC cells *in vivo* (Kubatka et al., 2016b; 2016a; 2017b; 2019; 2020a; 2020b; 2024). Small berries, such as seaberry, are recognized for their high antioxidant capacity, as indicated by ORAC rankings (Skrovankova et al., 2015; Wei et al., 2022). In this study, seaberry was evaluated for its effects on cellular malondialdehyde (MDA) levels, a biomarker of lipid peroxidation. The findings revealed that high-dose seaberry treatment significantly reduced MDA levels in BC cells compared to control samples. These results suggest that seaberry’s potent antioxidant activity, particularly its ability to protect genetic material from oxidative damage, represents a key mechanism underlying its anticancer/chemopreventive properties. In addition to antioxidative/genoprotective analyses, a study on *H. rhamnoides* fruit peels assessed their impact on MCF-7 and MDA-MB-231 BC cells, focusing on the mitigation of cisplatin cytotoxicity to evaluate protective potential. Pre-treatment with the SEAEtOH extract partially reduced cisplatin (IC_50_)-induced cytotoxicity in MCF-7 and MDA-MB-231 BC cells, indicating a potential genoprotective effect of the SEAEtOH extract. The antitumor properties of seaberry are primarily attributed to its antioxidant constituents, particularly phenolic compounds such as flavonoids (kaempferol, quercetin, catechin, and isorhamnetin) and carotenoids (lutein and zeaxanthin). These bioactive molecules mitigate oxidative damage, which can induce genetic mutations and contribute to carcinogenesis (Olas et al., 2018b). In this context, various plant-derived compounds have demonstrated genoprotective effects against oxidative and methylated DNA damage in human cancer cells (Thapa et al., 2019). Our findings indicate that the antioxidant potential of seaberry counteracted the cytotoxic effects of cisplatin, likely through free radical scavenging. Consequently, we emphasize the need for caution when combining potent natural antioxidants with alkylating agents such as cisplatin.

Phytochemicals exhibit significant antitumor activity, including pronounced effects on cancer stem cells (CSCs) (Oh et al., 2016; Chan et al., 2018; Liskova et al., 2019a; Liao et al., 2023). CSCs represent a specialized subpopulation of tumor cells with the capacity for self-renewal and differentiation into various lineages. They play a pivotal role in the multistage process of carcinogenesis, encompassing tumor initiation, promotion, progression, metastasis, and resistance to therapy (Huang et al., 2020). Clinically, CSCs are identified using well-established markers such as CD24, CD44, CD133, ALDH1, and EpCAM (Rabinovich et al., 2018; Zhang et al., 2020). In this study, high-dose seaberry treatment resulted in a notable reduction in the expression of CD44 and EpCAM compared to control groups. These findings align with our recent *in vivo* experiments in rat mammary carcinoma models, which demonstrated significant effects of plant-based foods such as sumac, salvia, and aronia on CSC markers (Kubatka et al., 2020b; 2024; Dvorska et al., 2024). Aronia treatment significantly reduced CD133 expression, while sumac showed a dose-dependent reduction in CD24, ALDH1, and EpCAM expression. Salvia decreased ALDH1 and EpCAM levels in BC cells. Similarly, in the same animal chemoprevention model, oregano, cloves, thyme, cinnamon, and sumac were observed to influence various CSC-related parameters (Kubatka et al., 2017a; 2017b; 2019; 2020a; 2020b; 2024; Dvorska et al., 2024). Our laboratory’s findings consistently demonstrate the beneficial effects of plant-based foods and phytochemicals on critical clinical markers of CSCs in BC. However, clinical research examining the impact of phytochemicals on CSCs significantly lags behind preclinical studies. Most clinical investigations prioritize the effects of synthetic drugs on CSC viability, leaving limited evidence on the role of plant-derived compounds as anti-CSC agents. Both our results and those from other researchers (Liskova et al., 2019b; Prajapati et al., 2022) emphasize the significant anti-CSC potential of phytochemicals across diverse cancer types. These effects are likely mediated through the modulation of multiple cell signaling pathways, underscoring the pressing need for comprehensive preclinical and clinical research in this domain (Dandawate et al., 2016).

The role of phytochemicals in modulating cancer-associated epigenetic mechanisms represents a critical area of investigation within oncological research. Epigenetic alterations involve modifications to global DNA methylation patterns, particularly in the promoter regions of oncogenes and tumor suppressor genes, as well as chemical changes to histones and the regulation of multiple genes by non-coding RNAs (Uramova et al., 2018; Tao et al., 2024). Various phytochemicals with antitumor activity have been shown to significantly affect the epigenetic landscape of neoplastic cells (Açar and Akbulut, 2023). Therefore, the effects of a phytochemical-rich diet on the cancer epigenome are of considerable clinical importance. Current research in oncology aims to identify the specific epigenetic changes induced by bioactive plant compounds (El Omari et al., 2021; Khan et al., 2024). Abnormal posttranslational histone modifications have been linked to the development of various chronic diseases and hold promise as reliable prognostic and predictive biomarkers in clinical settings, including oncology (Yang et al., 2022). Modulating histone-modifying enzymes through individual phytochemicals or their combinations may represent a novel therapeutic strategy for managing chronic diseases. In our chemopreventive study, seaberry dose-dependently increased H4K16ac and H4K20m3 levels in rat mammary tumor tissues. Similarly, beneficial histone modifications were observed following the long-term administration of clove buds (Kubatka et al., 2017b), thyme (Kubatka et al., 2019), cinnamon (Kubatka et al., 2020a), sumac (Kubatka et al., 2020b), salvia (Kubatka et al., 2024), and aronia (Dvorska et al., 2024) in the same BC model. In preclinical studies of other research groups, resveratrol has been reported to decrease H4R3me2s and H3K27me3 levels while increasing H3K9ac and H3K27ac levels in MCF-7 and MDA-MB-231 BC cell lines, effects linked to its cytotoxic activity and upregulation of tumor suppressor genes, including *BRCA1*, *p53*, and *p21* (Chatterjee et al., 2019). Similarly, an *in vitro* study demonstrated that combined treatment with sulforaphane and withaferin A reduced histone deacetylase (HDAC) levels in MCF-7 and MDA-MB-231 cells, with increased histone methylation associated with cytotoxicity, apoptosis, and cell cycle arrest (Royston et al., 2017). Sharma et Tollefsbol (Sharma and Tollefsbol, 2022) further evaluated the epigenetic effects of sulforaphane, genistein, and sodium butyrate in MCF-7 and MDA-MB-231 cells. Their findings revealed that dual and triple compound combinations more effectively downregulated HDACs (HDAC1, HDAC6, and HDAC11), histone methyltransferases (EZH2 and SUV39H1), and histone acetyltransferases (GCN5, PCAF, P300, and CBP) compared to single-agent treatments. These combinations induced global epigenetic alterations, including inhibition of HDAC activity, reduced histone H3 methylation at lysines 27 (H3K27me) and 9 (H3K9me), and increased histone acetyltransferase activity. While our study did not elucidate the precise mechanisms by which seaberry modulates posttranslational histone modifications in the *in vivo* BC model, extensive preclinical and clinical evidence highlights dietary phytochemicals as a promising strategy for cancer control (Uramova et al., 2018; Samec et al., 2019a). Gaining a deeper understanding of the molecular mechanisms underlying post-translational histone modifications could uncover novel therapeutic targets for plant-derived compounds, warranting further investigation.

MicroRNAs play a crucial role in regulating various cellular functions and gene expression during protein synthesis. Numerous phytochemicals have been identified as effective modulators of miRNA activity in carcinogenesis (Srivastava et al., 2015; Debnath et al., 2017; Samec et al., 2019b). In our study, an analysis of 758 miRNAs across 29 individual samples revealed significant alterations in the expression of nine miRNAs—miR-10a-5p, miR-322-5p, miR-450a-5p, miR-142-5p, miR-148b-3p, miR-1839-3p, miR-18a-5p, miR-1949, and miR-347—following seaberry administration in a rat chemoprevention model. In the context of BC research, miR-10a-5p, miR-322-5p, and miR-450a-5p have been identified as tumor suppressors (Khan et al., 2015; Ke and Lou, 2017; Rodriguez-Barrueco et al., 2017; Bieg et al., 2019; Zhang et al., 2022). Conversely, miR-142-5p and miR-148b-3p exhibit oncogenic potential (Dai et al., 2019; Yu et al., 2019; Chen et al., 2023; Hao et al., 2023), while miR-18a-5p has been reported to possess both oncogenic and tumor-suppressor properties in BC (Kolenda et al., 2020; Liu and Yang, 2023; Nair et al., 2024). To our knowledge, the roles of miR-1839-3p, miR-1949, and miR-347 in BC have not yet been characterized. Our findings highlight the significant beneficial effects of seaberry on miRNA expression, with no detectable adverse effects, in the assessment of a comprehensive panel of miRNAs in a rat model of BC. Recent studies of our group (Kubatka et al., 2019; 2020a; 2020b; 2024; Dvorska et al., 2024) analyzing reliable biomarkers for BC diagnosis and prognosis including oncogenic miR-21, miR-210, and miR-155, and tumor suppressors miR-22, miR-34a, and miR-145, revealed significant beneficial effects of *T. vulgaris*, *C. zeylanicum*, *R. coriaria*, *S. officinalis*, and *Aronia melanocarpa* L. on their expression in the same BC model. However, given the inconsistencies in data across broader BC research, including findings from our laboratory, there is a need for rigorous clinical and preclinical validation to better understand the molecular mechanisms. This will help clarify the role of specific miRNA signatures and individual miRNAs in BC and its subtypes, particularly in the context of diagnosis, prognosis, and prevention strategies (Arun et al., 2022; Verma et al., 2024).

The silencing of numerous cancer-associated genes is frequently attributed to methylation processes (Nishiyama and Nakanishi, 2021). Hyper-methylation of tumor suppressor gene (TSG) promoters represents a pivotal mechanism in oncogenesis. Conversely, hypomethylation of gene promoters generally correlates with increased gene expression (Ng and Yu, 2015). In BC patients and preclinical models, TSGs such as *ATM*, *PITX2*, *RASSF1A*, *PTEN*, and *TIMP3* are consistently found to exhibit reduced expression (Jasek et al., 2019). In this study, as well as in our prior extensive research with the same BC chemoprevention model, we analyzed the methylation patterns of CpG islands located within the promoter regions of TSGs in rat mammary carcinomas under chemopreventive conditions. Our previous work showed that plant-based bioactive compounds favorably alter TSG promoter methylation. Specifically, compounds from *Syzygium aromaticum*, *T. vulgaris*, *C. zeylanicum*, *R. coriaria*, *S. officinalis*, and *A. melanocarpa* were shown to significantly reduce methylation levels of clinically relevant TSG promoters. Targeted effects included *RASSF1* by clove; *ATM* by thyme; *ATM* and *TIMP3* by cinnamon; *ATM*, *PTEN*, and *TIMP3* by sumac; *ATM* and *PTEN* by sage; and *TIMP3* by aronia (Kubatka et al., 2017b; 2019; 2020a; 2020b; 2024; Dvorska et al., 2024). Notably, treatment with *H. rhamnoides* L. yielded a significant reduction in methylation of the *PITX2*, *RASSF1*, *PTEN*, and *TIMP3* promoters, except *ATM*. These findings, supported by other preclinical and clinical BC studies, underscore the epigenetic-modulating potential of plant-derived bioactive compounds. By influencing DNA methyltransferase activity, these compounds can reverse aberrant methylation patterns associated with BC development (Qin et al., 2009; 2014; Li et al., 2013; Wang et al., 2015; Jiang et al., 2016; Gao and Tollefsbol, 2018; Lewis et al., 2018; Nirgude et al., 2022; Aanniz et al., 2024). The modulation of TSG promoter methylation profiles highlights their potential as chemopreventive and therapeutic agents in BC. Future oncology research should prioritize translating these results into clinical settings, focusing on personalized approaches to leverage the epigenetic effects of plant-derived substances for BC prevention and treatment, particularly in high-risk populations (Jasek et al., 2019).

Although preliminary findings suggest that sea buckthorn exhibits anticancer potential, direct comparisons with established chemotherapeutic agents such as paclitaxel, doxorubicin, or tamoxifen remain scarce. However, investigations involving other bioactive natural compounds offer valuable insight. For instance, a study evaluating the efficacy of an edible seaweed extract *versus* tamoxifen in a rat model of BC reported superior tumor suppression and antioxidant effects with seaweed, alongside reduced hepatotoxicity and nephrotoxicity (Shamsabadi et al., 2013). Phytochemicals have been increasingly recognized for their potential to complement conventional chemotherapy by lowering the required drug dosage, thereby mitigating adverse effects and enhancing the sensitivity of chemoresistant cancer cells. This combinatorial strategy is gaining attraction in oncology research. Our recent preliminary *in vitro* investigations using MCF-7 and MDA-MB-231 BC cells demonstrated that sub-cytotoxic concentrations (IC_25_) of aronia extract, when combined with epirubicin, achieved comparable or enhanced inhibitory effects relative to epirubicin monotherapy (Dvorska et al., 2024). Notably, several phytochemical-drug combinations exhibited BC-resensitizing activities (Kang et al., 2009; Meng et al., 2016; Rad et al., 2025).

Incorporating plant-based nutraceuticals, such as seaberry, into BC therapy offers promise in enhancing therapeutic outcomes by resensitizing chemoresistant cancer cells and reducing chemotherapy-induced toxicity. However, comprehensive clinical investigations are essential to establish standardized treatment protocols and fully elucidate the potential benefits and risks associated with these integrative approaches.

## 5 Conclusion

Our study represents the first comprehensive preclinical investigation into the anti-cancer properties of *H. rhamnoides* (seaberry) fruit peels using BC animal models and *in vitro* approaches. The findings demonstrate significant anti-cancer effects of *H. rhamnoides* in two rodent BC models: an allograft mouse model and a chemically induced cancer chemoprevention model in rats. Mechanistic assessments revealed that seaberry fruit peels exert pro-apoptotic and antiproliferative effects through *in vivo* and *in vitro* experimental approaches. Furthermore, they exhibit antiangiogenic properties, enhance antioxidant activity, reduce cancer stemness, and induce favorable epigenetic modifications, including histone chemical alterations, miRNA regulation, and methylation of tumor suppressor gene (TSG) promoters in BC cells *in vivo*. These multi-level mechanisms contributed to a significantly improved tumor prognosis in the treated rodent models. The observed anticancer effects are linked to the activation of non-specific cellular signaling pathways, underscoring the need for further exploration of these networks in translational research.

Translating preclinical findings to human applications involves several challenges. Animal models often fail to fully replicate the genetic diversity and tumor microenvironment of human cancers, leading to discrepancies in treatment efficacy and safety during clinical transitions. A relatively high percentage of drugs that show promise in animal studies fail in human clinical trials, underscoring the predictive limitations of such models (Hackam and Redelmeier, 2006; Seyhan, 2019). Furthermore, combining plant-derived compounds like seaberry with conventional chemotherapy raises concerns about potential herb-drug interactions. Herbs contain biologically active compounds that can interfere with the metabolism of chemotherapy drugs, potentially reducing their efficacy or increasing toxicity (Ramos-Esquivel et al., 2017). Establishing appropriate dosage adjustments for seaberry-derived treatments is also critical. The bioavailability and metabolism of phytochemicals can vary significantly between species, and dosages effective in animal models may not directly translate to humans. Comprehensive pharmacokinetic studies are necessary to determine safe and effective dosing regimens for human patients (Nishimuta et al., 2013).

Despite the notable anti-cancer potential of plant-derived compounds and their natural combinations, including those in seaberry, their clinical application in BC treatment remains unrealized. Advancing their therapeutic potential requires addressing key challenges: (1) understanding pharmacokinetics to ensure safe and effective dosing, (2) developing advanced delivery systems such as nanoemulsions and nanoparticles for improved targeting and safety, (3) stratifying BC types using a multi-omics framework to align with individual patient characteristics, and (4) optimizing combinations with conventional chemotherapeutic agents to enhance efficacy.

Well-designed preclinical and clinical studies are essential for evaluating the efficacy, safety, and cost-effectiveness of plant nutraceuticals, such as seaberry, as potential agents for BC treatment and chemoprevention. Engaging in rigorous research will help bridge the gap between promising preclinical findings and practical clinical applications, ultimately improving patient outcomes.

## Data Availability

The raw data supporting the conclusions of this article will be made available by the authors, without undue reservation.

## Abbreviations

BC, breast carcinoma; CSC, cancer stem cell; DMSO, dimethyl sulfoxide; FC, fold-change; HDAC, *histone deacetylase;* HG/LG, high-/low-grade; LC-DAD-MS, liquid chromatography with diode array detection mass spectrometry; *ORAC,* oxygen radical absorbance capacity; ROS, reactive oxygen species; SEA, seaberry; *TSG, tumor-suppressor genes*.
