# Supplementary material for: Chemopreventive and therapeutic effects of Hippophae rhamnoides L. fruit peels evaluated in preclinical models of breast carcinoma
Source: Front Pharmacol. 2025 Apr 30;16:1561436. doi: 10.3389/fphar.2025.1561436 (PMC12075410; doi:10.3389/fphar.2025.1561436)
Supplement: Supplementary file 1 [file DataSheet1.docx]

**Supporting Information.**

**Chemopreventive and Therapeutic Effects of *Hippophae rhamnoides* L. Fruit Peels Evaluated in Preclinical Models of Breast Carcinoma**

Dana Dvorska^1^, Dominika Sebova^2^, Karol Kajo^3^, Andrea Kapinova^1^, Emil Svajdlenka^4^, Michal Goga^5^, Richard Frenak^5^, Jakub Treml^6^, Sandra Mersakova^1^, Jan Strnadel^1^, Alena Mazurakova^7^, Ivana Baranova^8,9^, Erika Halasova^1^, Mariana Brozmanová^8^, Kamil Biringer^10^, Monika Kassayová^11^, Zuzana Dankova^1,9^, Karel Smejkal^4^, Slavomir Hornak^12^, Jan Mojzis^2^, Vladimira Sadlonova^13^, Dusan Brany^1,*^, Martin Kello^2,*^, Peter Kubatka^14,*^

^1^Biomedical Centre Martin, Jessenius Faculty of Medicine, Comenius University in Bratislava, Mala Hora 4D, 03601 Martin, Slovakia; [dana.dvorska@uniba.sk](mailto:dana.dvorska@uniba.sk) (D.D.); [sandra.mersakova@uniba.sk](mailto:sandra.mersakova@uniba.sk) (S.M.); [jan.strnadel@uniba.sk](mailto:jan.strnadel@uniba.sk) (J.S.); [erika.halasova@uniba.sk](mailto:erika.halasova@uniba.sk) (E.H.); zuzana.[dankova@uniba.sk](mailto:dankova@uniba.sk) (Z.D.); [dusan.brany@uniba.sk](mailto:dusan.brany@uniba.sk) (D.B.)

^2^Department of Pharmacology, Faculty of Medicine, P. J. Šafárik University, 040 11 Košice, Slovakia; [dominika.sebova@student.upjs.sk](mailto:dominika.sebova@student.upjs.sk) (D.S.); [jan.mojzis@upjs.sk](mailto:jan.mojzis@upjs.sk) (J.M.); [martin.kello@upjs.sk](mailto:martin.kello@upjs.sk) (M.K.)

^3^Department of Pathology, St. Elisabeth Oncology Institute, 812 50 Bratislava, Slovakia; kkajo[@ousa.sk](mailto:dev@null) (K.K.)

^4^Department of Natural Drugs, Faculty of Pharmacy, Masaryk University, 612 00 Brno, Czech Republic; svajdlenka@pharm.muni.cz (E.S.); [smejkalk](mailto:smejkalk)@pharm.muni.cz (K.S.)

^5^Department of Botany, Institute of Biology and Ecology, Faculty of Science, P. J. Safarik University, 04001 Kosice, Slovakia, [michal.goga@upjs.sk](mailto:michal.goga@upjs.sk) (M.G.); [richard.frenak@student.upjs.sk](mailto:richard.frenak@student.upjs.sk) (R.F.)

^6^Department of Molecular Pharmacy, Faculty of Pharmacy, Masaryk University, 612 00 Brno, Czech Republic; [tremlj@pharm.muni.cz](mailto:tremlj@pharm.muni.cz) (J.T.)

^7^Department of Anatomy, Jessenius Faculty of Medicine in Martin, Comenius University in Bratislava, 03601 Martin, Slovakia, [alena.mazurakova@uniba.sk](mailto:alena.mazurakova@uniba.sk) (A.M.)

^8^Department of Pathological Physiology, Jessenius Faculty of Medicine, Comenius University in Bratislava, Martin, Slovakia; [ivana.baranova@uniba.sk](mailto:ivana.baranova@uniba.sk) (I.B.); [mariana.brozmanova@uniba.sk](mailto:mariana.brozmanova@uniba.sk) (M.B.)

^9^Biobank for Cancer and Rare Diseases, Jessenius Faculty of Medicine, Comenius University in Bratislava, Martin, Slovakia

^10^Clinic of Obstetrics and Gynecology, Jessenius Faculty of Medicine, Comenius University in Bratislava, 03659 Martin, Slovakia, [kamil.biringer@uniba.sk](mailto:kamil.biringer@uniba.sk) (K.B.)

^11^Department of Animal Physiology, Institute of Biology and Ecology, Faculty of Science, P. J. Safarik University, 04001 Kosice, Slovakia, [monika.kassyova@upjs.sk](mailto:monika.kassyova@upjs.sk) (M.K.)

^12^Small Animal Clinic, University of Veterinary Medicine and Pharmacy, 041 81 Kosice, Slovakia, [slavomir.hornak@uvlf.sk](mailto:slavomir.hornak@uvlf.sk) (S.H.)

^13^Department of Microbiology and Immunology, Jessenius Faculty of Medicine, Comenius University in Bratislava, Martin, Slovakia; [vladimira.sadlonova@uniba.sk](mailto:vladimira.sadlonova@uniba.sk) (V.S.)

^14^Centre of Experimental and Clinical Regenerative Medicine, Small Animal Clinic, University of Veterinary Medicine and Pharmacy, 041 81 Kosice, Slovakia, [peter.kubatka@uvlf.sk](mailto:peter.kubatka@uvlf.sk) (P.K.)

 Figure S1. HPLC-DAD of ethanol-water extract of fruit peel of *H. rhamnoides*, quercetin 9.57 min., kaempferol 11.40 min., isorhamnetin 11.70 min. and internal standard chrysin 14.32 min., 465-475 nm.

Figure S2. HPLC-MS/MS of ethanol-water extract of fruit peel of *H. rhamnoides*, 10× diluted with ethanol, catechin (blue and red) and EGCG (green and gray)

Figure S3. HPLC-DAD of hexane extract of fruit peel of *Hippophae rhamnoides* L., 446-454 nm
